# Microbial CO_2_ bioconversion: integrating carbon fixation pathways and sustainable biomanufacturing

**DOI:** 10.3389/fmicb.2026.1961758

**Published:** 2026-10-21

**Authors:** Elison de Souza Sevalho, Desireé Soares da Silva, Kamila de Sousa Gomes, Leandro da Conceição, Layssa Aline Okamura, Jessica Carolina Medina-Gallardo

**Affiliations:** Instituto SENAI de Inovação em Biomassa (ISI Biomassa), Serviço Nacional de Aprendizagem Industrial, Três Lagoas, Brazil

**Keywords:** carbon capture, utilization and storage, carbon fixation pathways, circular bioeconomy, metabolic engineering, microbial electrosynthesis, synthetic biology

## Abstract

The continued rise in atmospheric carbon dioxide (CO_2_) concentrations has intensified the need for sustainable technologies capable of mitigating greenhouse gas emissions while generating value-added products. In this context, microbial CO_2_ bioconversion has emerged as a promising biological strategy within broader carbon capture, utilization, and storage (CCUS) frameworks, particularly through biological carbon capture and utilization (bio-CCU). These approaches enable the conversion of inorganic carbon into fuels, chemicals, biomaterials, proteins, and other value-added products. This review provides a comprehensive assessment of recent advances in microbial CO_2_ bioconversion by bridging biological, technological, and industrial perspectives. Unlike previous reviews focusing on individual topics, such as carbon fixation pathways, gas fermentation, or metabolic engineering, this work presents an integrated framework connecting natural and synthetic carbon fixation pathways, microbial production platforms, metabolic engineering strategies, product diversification, and techno-economic considerations. To support this analysis, we conducted a systematic bibliometric assessment of 913 publications indexed in the Web of Science Core Collection, revealing sustained growth in scientific output, increasing interdisciplinarity, structured collaboration networks, and strong thematic interconnections across microbial ecology, carbon fixation, metabolism, and carbon utilization. Recent advances in synthetic biology, systems-level metabolic engineering, microbial electrosynthesis, bioelectrochemical systems, synthetic microbial consortia, and artificial intelligence-assisted strain engineering are discussed as important drivers of microbial carbon utilization. The review also critically examines major barriers to industrial implementation, including CO_2_ mass transfer, carbon fixation efficiency, energy constraints, process scale-up, downstream processing, and economic feasibility. By integrating systematic bibliometric evidence with a critical assessment of microbial CO_2_ biomanufacturing, this review identifies the convergence of microbial physiology, metabolic engineering, bioprocess development, renewable energy, and data-driven approaches as central to advancing biological CO_2_ conversion toward scalable, sustainable biomanufacturing.

## 1 Introduction

The sustained accumulation of atmospheric greenhouse gases (GHGs) has intensified global environmental challenges, including climate change, global warming, ocean acidification, sea-level rise, and the increasing frequency of extreme weather events. Among these gases, carbon dioxide (CO_2_) remains the principal anthropogenic contributor to global warming, primarily due to the persistent dependence on fossil fuels and land-use changes (Jones et al., 2023; Lamb et al., 2024; Robiou du Pont et al., 2025). Despite decades of international climate initiatives under the United Nations Framework Convention on Climate Change (UNFCCC), the Kyoto Protocol, and the Paris Agreement, fossil fuels continue to supply more than 80% of global primary energy demand and account for nearly 68% of anthropogenic greenhouse gas emissions (Achakulwisut et al., 2023; Portillo Juan et al., 2024; Liang et al., 2025). According to the World Meteorological Organization (WMO), the global atmospheric CO_2_ concentration reached 423.9 ppm in 2024, representing the highest level observed in at least the past 800,000 years and highlighting the urgency of developing effective carbon mitigation strategies (Krapp et al., 2021; World Meteorological Organization [WMO], 2024; Su et al., 2025).

Growing scientific evidence indicates that emission reduction alone will be insufficient to achieve the carbon neutrality targets established by most countries. Consequently, nearly 200 nations have incorporated carbon mitigation commitments into their Nationally Determined Contributions (NDCs), while major economies including the European Union, the United States, Japan, and China have adopted long-term decarbonization strategies aimed at achieving net-zero emissions between 2050 and 2060 (Zhong et al., 2025; Albanese et al., 2025; Guo et al., 2025). However, the continued accumulation of atmospheric CO_2_ demonstrates that additional technologies capable of removing, converting, and valorizing carbon emissions are essential to complement conventional mitigation measures (Begum and Mobin, 2025).

In this context, carbon capture, utilization, and storage (CCUS) has emerged as one of the most promising technological frameworks for reducing emissions from hard-to-abate sectors such as cement, steel, chemicals, and aviation. While renewable energy deployment and energy-efficiency improvements remain fundamental pillars of climate mitigation, CCUS technologies provide complementary solutions by enabling the capture and conversion of unavoidable CO_2_ emissions into value-added products. Conventional post-combustion capture technologies, particularly solvent-based chemical absorption, are already commercially deployed at large scales. Nevertheless, their high energy demand, solvent degradation, corrosion, and operational costs continue to motivate the development of alternative carbon utilization strategies with improved sustainability and economic performance (Chaturwedi et al., 2025).

Among these alternatives, biological CO_2_ capture and utilization (bio-CCU) has attracted increasing attention because it combines carbon mitigation with the sustainable production of fuels, chemicals, food ingredients, polymers, and advanced biomaterials. Unlike purely physicochemical conversion processes, microbial platforms operate under mild reaction conditions, exhibit high product selectivity, tolerate impurities commonly present in industrial gas streams, and can synthesize structurally complex molecules that remain difficult to obtain through conventional catalytic routes (Li et al., 2020). These characteristics position microbial systems as important complementary technologies within future integrated carbon management strategies rather than direct replacements for thermochemical or electrochemical processes.

Microorganisms have performed biological CO_2_ fixation for billions of years through diverse metabolic pathways that convert inorganic carbon into biomass and reduced carbon compounds. Recent advances in synthetic biology, systems metabolic engineering, genome editing, adaptive laboratory evolution, and systems biology have substantially expanded the capabilities of microbial platforms. Engineered microorganisms can now incorporate optimized carbon-fixation pathways, improve intracellular redox balance, enhance carbon assimilation efficiency, and produce an increasingly diverse portfolio of fuels, organic acids, alcohols, polymers, proteins, and other high-value bioproducts from CO_2_ (Della Valle et al., 2024; Lee et al., 2025). Simultaneously, emerging technologies such as gas fermentation, microbial electrosynthesis, hybrid electro-biological systems, and artificial carbon-fixation pathways are accelerating the transition of microbial CO_2_ biotransformation from laboratory research toward industrial application (Cui et al., 2025).

Despite these remarkable advances, the field has expanded rapidly across multiple disciplines including microbiology, metabolic engineering, electrochemistry, reactor engineering, synthetic biology, systems biology, and industrial biotechnology, resulting in an increasingly fragmented body of literature. Existing reviews have generally focused on individual topics, such as carbon-fixation pathways, gas fermentation, microbial electrosynthesis, metabolic engineering, or specific classes of CO_2_-derived products. Consequently, relatively few studies have provided an integrated perspective that simultaneously addresses microbial physiology, natural and synthetic carbon-fixation pathways, emerging bioconversion technologies, product diversification, industrial implementation, techno-economic feasibility, life cycle assessment, and future technological directions. This fragmentation hampers the identification of research priorities and limits a systems-level understanding of microbial CO_2_ biomanufacturing.

Therefore, this review provides a comprehensive and integrated assessment of recent advances in microbial CO_2_ biotransformation, encompassing natural and synthetic carbon-fixation pathways, microbial conversion platforms, metabolic engineering strategies, and the expanding portfolio of CO_2_-derived bioproducts. In addition to summarizing current knowledge, the review critically compares biological conversion routes, discusses their technological maturity and industrial applicability, evaluates the major scientific, engineering, and economic bottlenecks that remain, and highlights emerging opportunities for developing scalable and economically viable microbial platforms capable of supporting a future low-carbon bioeconomy.

## 2 Literature search methodology and bibliometric analysis

### 2.1 Review design and reporting framework

This study was conducted as a systematic scoping review integrated with bibliometric analysis to evaluate the scientific development, technological evolution, and emerging trends in microbial CO_2_ bioconversion. The review was reported in accordance with the Preferred Reporting Items for Systematic Reviews and Meta-Analyses (PRISMA 2020) framework and the PRISMA extension for Scoping Reviews (PRISMA-ScR), where applicable. The bibliometric component was adapted from the methodological framework proposed by de Souza Sevalho et al. (2023) and integrated with systematic screening and critical synthesis of the retrieved literature. The study selection process is presented in the PRISMA 2020 flow diagram (Figure 1), while the complete search strategy, screening decisions, and eligibility information are provided in Supplementary Data 1.

**FIGURE 1:**
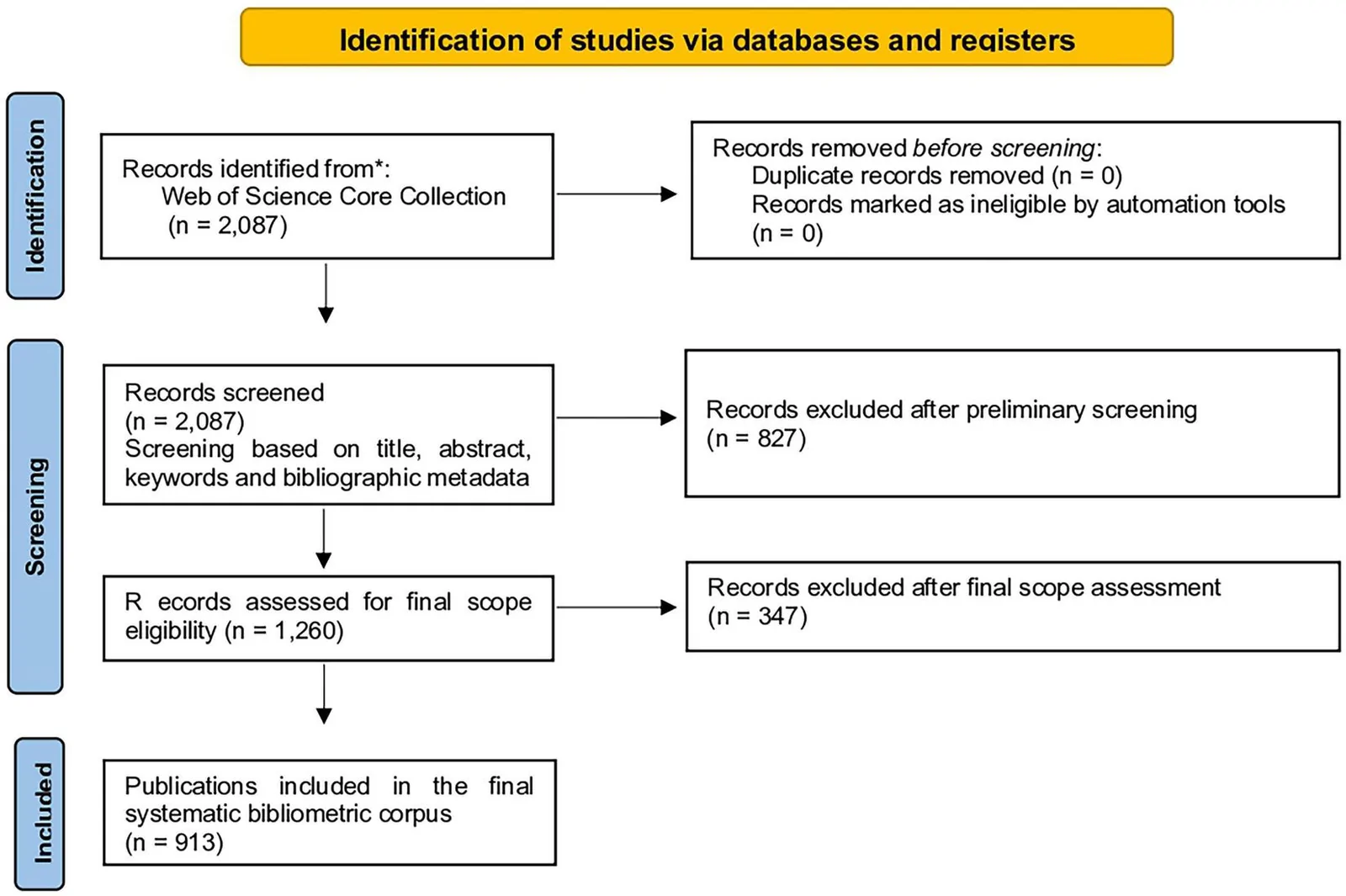
Study identification, screening, and selection process for the systematic bibliometric analysis of microbial CO_2_ bioconversion research. The PRISMA 2020 flow diagram summarizes the identification and screening of records retrieved from the Web of Science Core Collection. A total of 2,087 records were initially identified and screened based on title, abstract, keywords, and bibliographic metadata. After preliminary screening, 827 records were excluded, leaving 1,260 records for final scope assessment. An additional 347 records were excluded following the final eligibility assessment, resulting in 913 publications included in the final systematic bibliometric corpus.

Given the scoping and evidence-mapping nature of this review, the conventional intervention-based PICOS framework was not directly applicable. Accordingly, the review question was structured around the microbial systems investigated, CO_2_ fixation and bioconversion strategies, metabolic and technological approaches, resulting products and processes, and their relevance to sustainable biomanufacturing.

### 2.2 Information source and search strategy

The literature search was conducted in the Web of Science (WoS) Core Collection (Clarivate Analytics, Philadelphia, PA, USA), on August 10, 2026, covering publications indexed from 2010 up to the date of the database search. Records published in 2026 were included when already available and indexed in the database at the time of retrieval. This time frame was selected to capture the marked scientific and technological expansion of microbial CO_2_ bioconversion, encompassing advances in synthetic biology, metabolic engineering, gas fermentation, microbial electrosynthesis, and carbon capture and utilization technologies.

The search strategy was designed to identify publications addressing microbial CO_2_ fixation, biotransformation, and biotechnological applications involving bacterial carbon conversion. The search was performed using the Smart Search interface of the Web of Science Core Collection, applying the following query: (“CO_2_ biotransformation” OR “carbon dioxide utilization” OR “CO_2_ conversion” OR “carbon fixation”) AND (“bacteria” OR “bacterial” OR “autotrophic bacteria”). The retrieved records were subsequently subjected to the predefined database refinement and eligibility procedures described below. The complete search strategy, bibliographic metadata, screening decisions, and final composition of the corpus are provided in Supplementary Data 1, ensuring transparency and traceability of the study-selection process.

### 2.3 Eligibility criteria

Records were considered eligible when they addressed microbial CO_2_ fixation, assimilation, biotransformation, or CO_2_-based biotechnological processes and provided information relevant to microbial physiology, carbon-fixation pathways, metabolic or genetic mechanisms, bioprocess development, microbial electrosynthesis, gas fermentation, or CO_2_-derived bioproducts. Records addressing exclusively physicochemical CO_2_ capture, geological storage, abiotic conversion, environmental carbon cycling without a direct biotechnological component, or processes without clear microbial involvement were excluded. Records whose scope remained ambiguous after preliminary screening were subjected to additional scope assessment. Eligible records encompassed experimental studies and other peer-reviewed research articles providing biological, metabolic, technological, or bioprocess evidence relevant to the scope of the review.

### 2.4 Study selection and screening

All records retrieved from the Web of Science Core Collection were screened using predefined eligibility criteria. No duplicate records were removed prior to the screening stage. Screening was based on titles, abstracts, keywords, and available bibliographic metadata. Records showing a clear lack of alignment with microbial CO_2_ fixation, bioconversion, or related biotechnological applications were excluded during the preliminary screening stage. Records requiring further assessment were subjected to a more detailed scope evaluation based on the same predefined criteria.

The screening and eligibility assessment were conducted by members of the review team. Records with uncertain eligibility were jointly reassessed, and final inclusion or exclusion decisions were established by consensus. The complete screening decisions and exclusion criteria are provided in Supplementary Data 1. The study selection process is reported using the PRISMA 2020 flow diagram (Figure 1).

### 2.5 Data extraction and qualitative synthesis

Bibliographic metadata and thematic information relevant to the objectives of the review were extracted and organized from the eligible literature. The qualitative synthesis considered microbial taxa, carbon-fixation pathways, metabolic routes, key enzymes and genes, bioreactor configurations, operational conditions, CO_2_ conversion strategies, conversion performance, and reported bioproducts. This information supported the systematic qualitative synthesis and thematic organization of the review. Given the evidence-mapping scope of the review, the absence of intervention-effect synthesis, and the methodological heterogeneity of the included literature, a conventional study-level risk-of-bias assessment was not considered applicable. Instead, methodological transparency was supported through predefined eligibility criteria, systematic screening, documentation of exclusion decisions, and traceable bibliographic data extraction.

### 2.6 Bibliometric analysis

Bibliometric analyses were performed using the final corpus of 913 publications. Bibliographic metadata were structured to characterize temporal publication trends, contributing countries and institutions, disciplinary categories, and leading publication venues; the corresponding processed bibliometric dataset is provided in Supplementary Data 2.

Network analyses were performed using VOSviewer^®^ version 1.6.20 (Center for Science and Technology Studies, Leiden University, The Netherlands). Co-authorship analysis was used to evaluate collaboration patterns among authors, whereas keyword co-occurrence analysis was applied to characterize the major thematic structures of the research field. For the co-authorship network, fractional counting was applied, and authors meeting the predefined minimum publication threshold were retained. For keyword co-occurrence analysis, a minimum threshold of 20 occurrences was applied for keyword inclusion. Network relationships were normalized using the association-strength method, enabling visualization of collaboration structures, thematic clusters, and the strength of relationships among bibliometric items. The dataset used for VOSviewer network construction is provided in Supplementary Data 3.

### 2.7 Integration of systematic and bibliometric evidence

The bibliometric findings were integrated with the qualitative synthesis of the systematically selected evidence to identify publication trends, collaboration networks, technological advances, emerging research fronts, and persistent knowledge gaps. This integrated approach provided the framework for evaluating microbial carbon-fixation pathways, CO_2_ bioconversion technologies, bioproduct development, industrial translation, and future research directions.

## 3 Results and discussion

### 3.1 Bibliometric analysis of microbial CO_2_ bioconversion research

A bibliometric analysis was conducted using the Web of Science Core Collection to characterize the evolution and current scientific landscape of microbial CO_2_ bioconversion between 2010 and 2026. Following systematic screening and scope assessment, 913 publications were retained in the final bibliometric corpus (Figure 1). The analysis integrated temporal publication trends, geographical and institutional contributions, disciplinary distribution, publication venues, co-authorship structure, and keyword co-occurrence. Network analyses generated using VOSviewer were further employed to characterize collaboration patterns and the thematic organization of the field. Together, these analyses provide an overview of the scientific development, interdisciplinary expansion, and progressive technological diversification of microbial CO_2_ bioconversion research.

The systematic selection process substantially refined the initial literature dataset. A total of 2,087 records were identified from the Web of Science Core Collection, with no duplicate records removed before screening. Preliminary screening based on titles, abstracts, keywords, and bibliographic metadata excluded 827 records, leaving 1,260 publications for final scope assessment. A further 347 records were excluded because they did not meet the final eligibility criteria, resulting in 913 publications included in the systematic bibliometric corpus (Figure 1). This screening strategy ensured that the subsequent analyses were focused specifically on studies relevant to microbial CO_2_ bioconversion rather than the broader literature on carbon cycling, environmental microbiology, or non-biological carbon conversion.

As shown in Figure 2, the scientific literature exhibited a marked long-term expansion over the analyzed period. Annual publication output increased progressively, with a particularly pronounced acceleration during the most recent years, reaching its highest complete-year value in 2025 (*n* = 104). This trajectory reflects more than an increase in scientific output; it highlights the growing recognition of CO_2_ as a potential renewable carbon feedstock and microorganisms as versatile platforms for carbon capture and utilization, sustainable biomanufacturing, and circular bioeconomy strategies. Advances in synthetic biology, metabolic engineering, genome editing, renewable-energy integration, and microbial electrochemical technologies have progressively expanded the technological possibilities for biological CO_2_ conversion.

**FIGURE 2:**
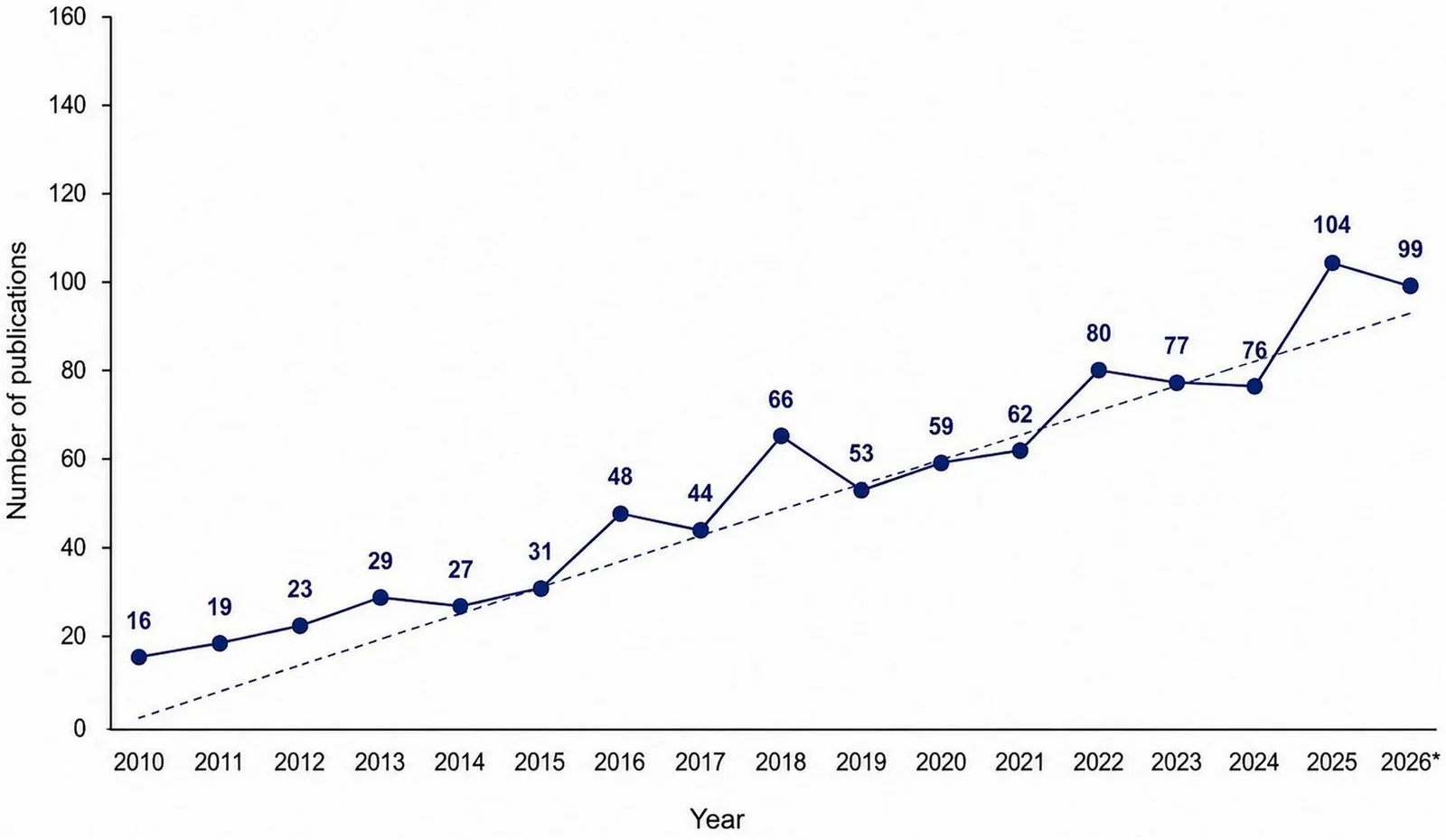
Temporal evolution of scientific publications on microbial CO_2_ bioconversion from 2010 to 2026. Annual publication output was determined from the final bibliometric corpus comprising 913 publications retrieved from the Web of Science Core Collection. The dashed line represents the overall temporal trend, evidencing the progressive expansion of research activity over the analyzed period. Publication output reached its highest value in 2025 (*n* = 104). Data for 2026 (*n* = 99*) correspond only to records available and indexed at the time of database retrieval and therefore represent a partial publication year.

Importantly, 99 publications were already indexed for 2026 at the time of database retrieval. Because this value represents only the records available at the time of data collection, it should be interpreted as a partial-year value rather than directly compared with complete annual outputs. Nevertheless, the high number of publications already recorded in 2026 reinforces the continued expansion of scientific activity in this area. Collectively, these temporal trends indicate that microbial CO_2_ bioconversion is evolving from a specialized research topic toward a multidisciplinary field positioned at the interface of microbiology, biotechnology, environmental science, carbon management, and sustainable biomanufacturing.

The geographical distribution of publications (Figures 3A,B) reveals a marked concentration of research activity in China and the United States, which represent the major contributors to the scientific literature on microbial CO_2_ bioconversion. Their prominence is consistent with substantial research investment in biotechnology, carbon capture and utilization, renewable energy, environmental technologies, and decarbonization strategies. Other countries, including Germany, Australia, Japan, and several European nations, also contribute relevant publication portfolios, demonstrating the increasingly international character of the field.

**FIGURE 3:**
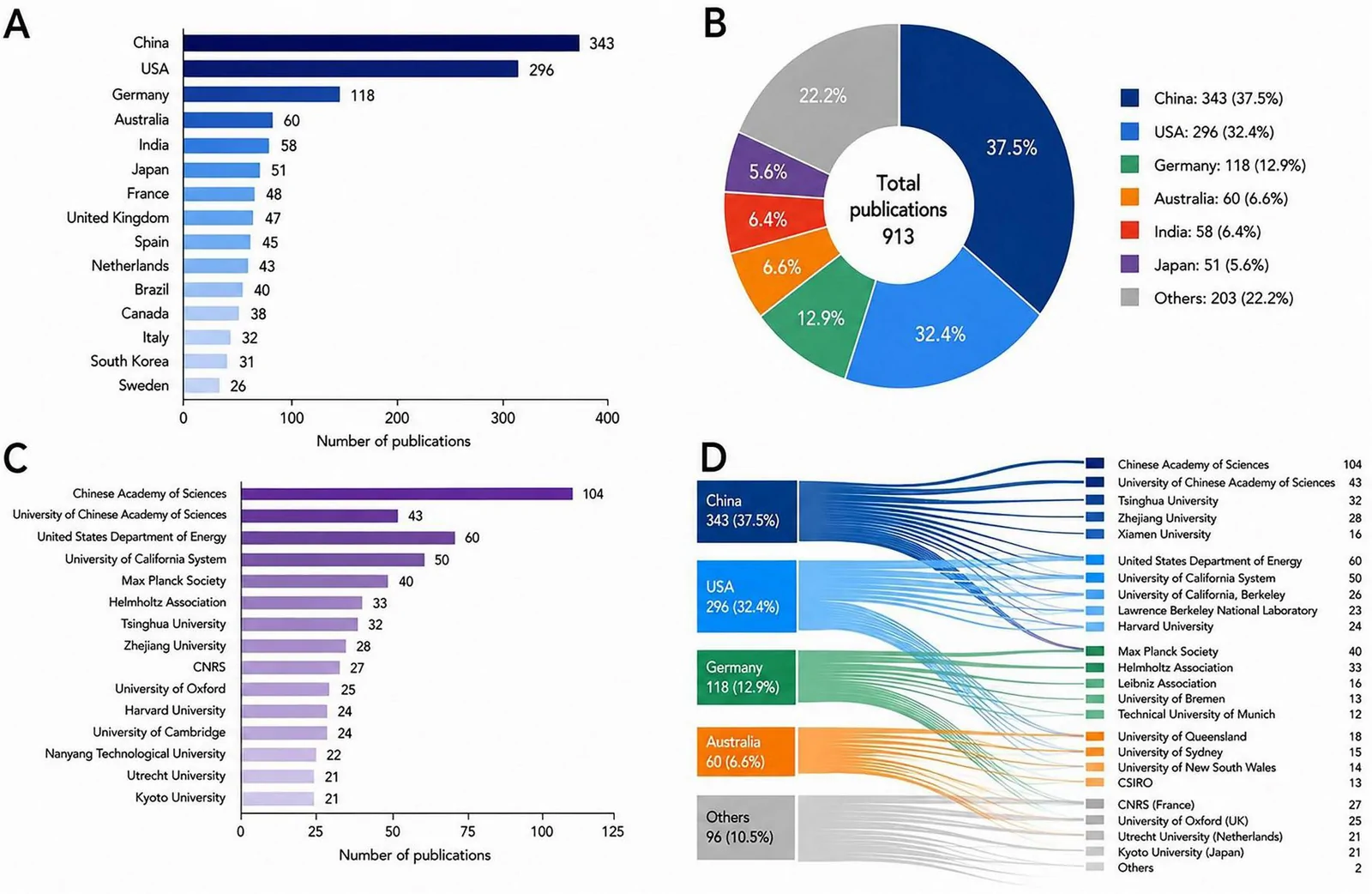
Global research landscape of microbial CO_2_ bioconversion. **(A)** Leading contributing countries ranked by number of publications. **(B)** Relative contribution of the leading countries to the final bibliometric corpus (*n* = 913). **(C)** Leading institutions ranked by publication output. **(D)** Sankey diagram showing the relationships between major contributing countries and their leading affiliated institutions. Country- and institution-level counts represent affiliation occurrences rather than mutually exclusive publications. Because a single publication may include authors from multiple countries and institutions, cumulative counts may exceed the total number of publications in the final bibliometric corpus (*n* = 913).

However, publication volume alone should not be interpreted as a direct measure of technological leadership or commercial maturity. Translation of microbial CO_2_ conversion technologies toward industrial implementation additionally depends on factors such as technology transfer, pilot-scale validation, intellectual property development, regulatory frameworks, access to renewable energy, industrial infrastructure, and private-sector investment. Therefore, the geographical distribution observed here primarily reflects the concentration and international distribution of scientific activity rather than industrial readiness.

A similar pattern is observed at the institutional level (Figures 3C,D), where publication output is concentrated within a relatively limited group of major research organizations. Chinese Academy of Sciences emerges as the leading institution, accompanied by prominent U.S. organizations, including the U.S. Department of Energy and the University of California system. The country–institution relationships illustrated by the Sankey diagram further demonstrate the contribution of major research organizations to national publication output.

This institutional concentration is consistent with the multidisciplinary requirements of microbial CO_2_ bioconversion, which increasingly combines microbial physiology, metabolic engineering, synthetic biology, reactor engineering, electrochemistry, computational approaches, and analytical technologies. Large research organizations with access to multidisciplinary infrastructure and long-term funding are therefore particularly well positioned to develop integrated research programs connecting fundamental microbial carbon metabolism with applied carbon-conversion technologies.

The disciplinary distribution of publications (Figure 4A) further demonstrates the interdisciplinary character of the field. Microbiology was the dominant Web of Science category (*n* = 599), followed by Environmental Sciences (*n* = 329) and Biotechnology & Applied Microbiology (*n* = 235). Relevant contributions were also observed for Science & Technology – Other Topics (*n* = 173), Ecology (*n* = 160), Engineering (*n* = 93), Marine & Freshwater Biology (*n* = 59), Energy & Fuels (*n* = 44), Water Resources (*n* = 41), and Agriculture (*n* = 38).

**FIGURE 4:**
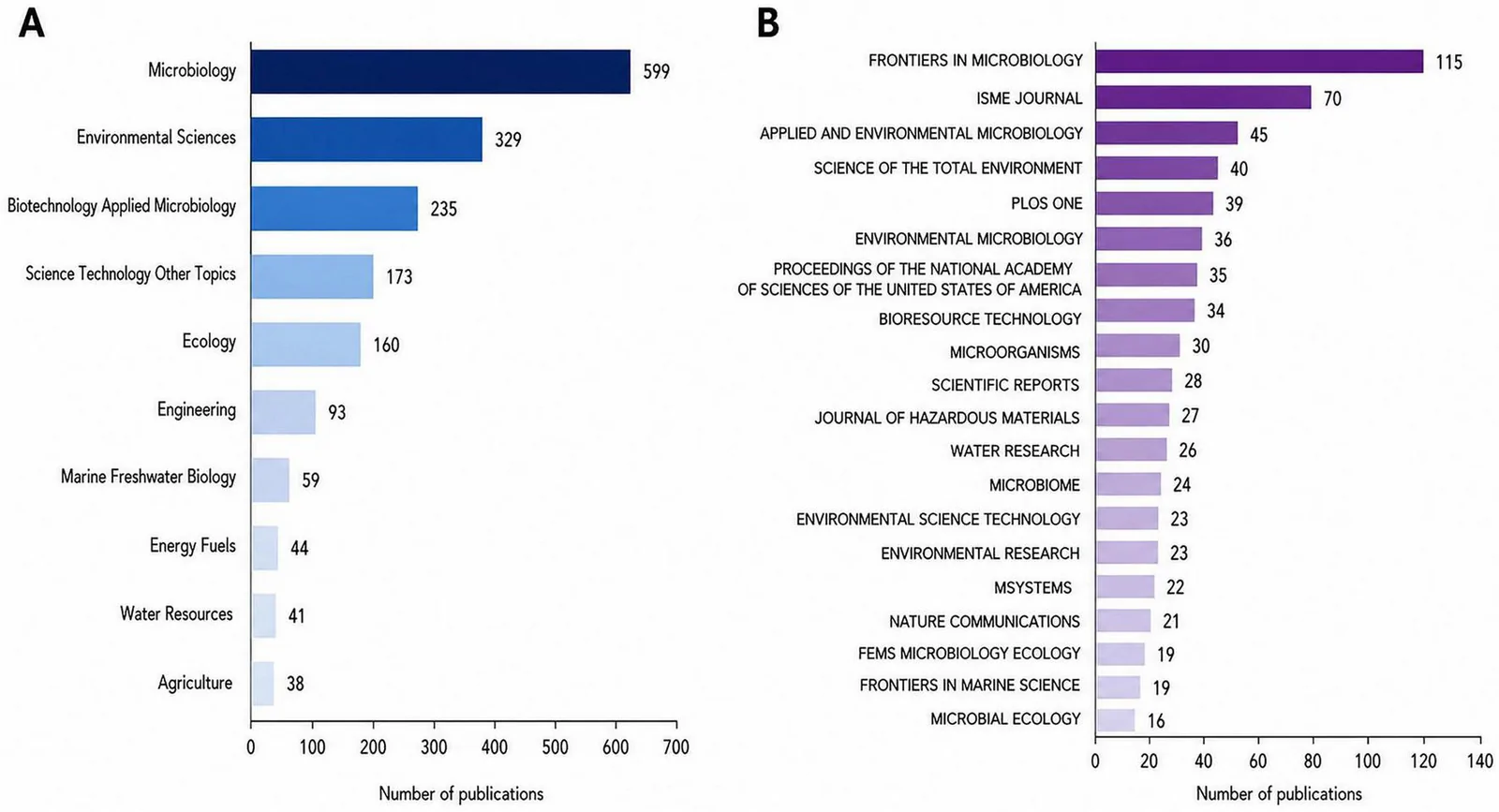
Disciplinary distribution and leading publication venues in microbial CO_2_ bioconversion research. **(A)** Distribution of the final bibliometric corpus across the leading Web of Science Categories. **(B)** Leading source journals ranked by number of publications. Publications may be assigned to more than one Web of Science category. Therefore, category counts represent non-mutually exclusive occurrences and the cumulative number of category assignments may exceed the total number of publications in the final bibliometric corpus (*n* = 913). Journal counts, in contrast, correspond to individual source titles within the final corpus.

The predominance of microbiology confirms that microbial physiology, metabolism, and carbon fixation remain the fundamental biological basis of CO_2_ bioconversion research. At the same time, the substantial contributions from environmental sciences, biotechnology, engineering, energy, and water-related disciplines demonstrate that the field increasingly extends beyond fundamental microbiology toward environmental and industrial applications. Because individual publications may be assigned to multiple Web of Science categories, these values represent non-mutually exclusive disciplinary occurrences rather than independent subsets of the 913-publication corpus.

Analysis of publication venues (Figure 4B) reinforces this interdisciplinary profile. Frontiers in Microbiology was the leading publication venue, with 115 publications, followed by ISME Journal (*n* = 70) and Applied and Environmental Microbiology (*n* = 45). Other prominent journals included Science of the Total Environment (*n* = 40), PLOS ONE (*n* = 39), Environmental Microbiology (*n* = 36), Proceedings of the National Academy of Sciences of the United States of America (*n* = 35), and Bioresource Technology (*n* = 34).

The strong representation of microbiology and microbial ecology journals demonstrates that biological mechanisms and microbial community functions remain central to the field. Conversely, the presence of environmental, biotechnology, water, and multidisciplinary journals reflects the increasing connection of microbial CO_2_ research with environmental engineering, resource recovery, carbon management, and sustainable bioprocess development. This publication pattern therefore supports a broader transition from predominantly descriptive studies of microbial carbon metabolism toward increasingly application-oriented research.

The co-authorship network provides a complementary perspective on the organization of the scientific community (Figure 5). Using the established minimum publication threshold, the final network comprised 85 authors distributed across 12 collaboration clusters, interconnected through 228 links and a total link strength of 137.50. The network reveals several recognizable research communities, with highly connected authors occupying central or bridging positions and smaller groups occurring toward the network periphery.

**FIGURE 5:**
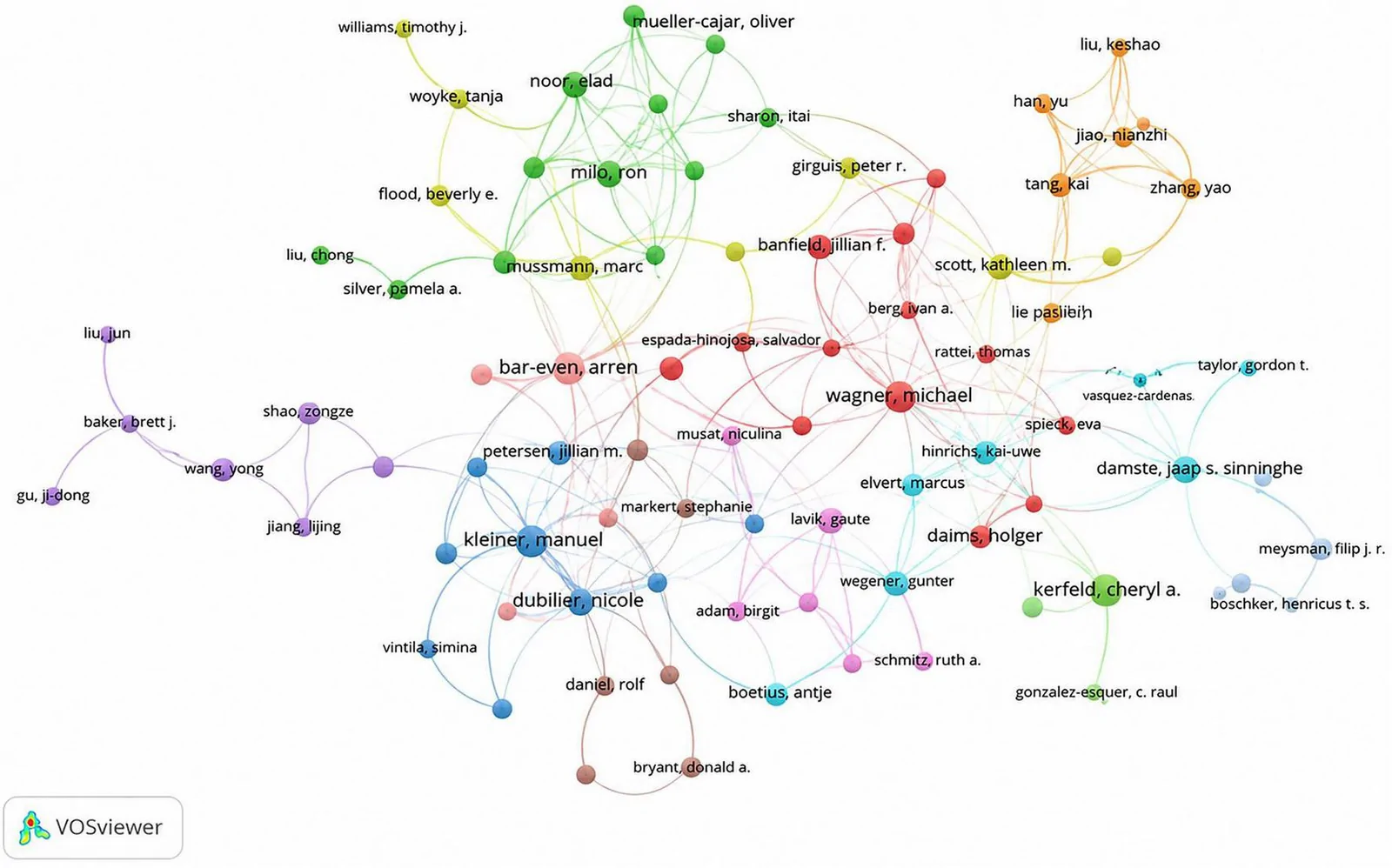
Global co-authorship network and collaboration structure in microbial CO bioconversion research. The network represents authors meeting the predefined minimum publication threshold using fractional counting. Node size is proportional to publication output, links represent co-authorship relationships, and colors indicate collaboration clusters identified by VOSviewer. The network comprised 85 authors distributed across 12 clusters, with 228 links and a total link strength of 137.50, providing a visual representation of the principal collaborative communities and connectivity patterns within the field.

Although several clusters display substantial internal connectivity, the persistence of distinct collaboration communities indicates that the global research landscape remains partially fragmented. Connections among clusters are present but less extensive than collaboration within individual research groups. This structure likely reflects the organization of the field around established laboratories, institutional partnerships, and specialized research programs. Greater interaction among these communities could facilitate the integration of complementary expertise in microbial physiology, synthetic biology, metabolic engineering, environmental biotechnology, electrochemistry, computational biology, and bioprocess engineering. Such integration is particularly relevant as microbial CO_2_ bioconversion advances toward more complex and scalable technological platforms.

The keyword co-occurrence analysis provides a broader representation of the conceptual structure of the literature (Figure 6). Applying a minimum occurrence threshold of 20 resulted in a final network containing 57 keywords organized into four thematic clusters, with 1,081 links and a total link strength of 1,084.00. The exceptionally high connectivity relative to the number of retained keywords demonstrates substantial conceptual overlap among the major research themes and suggests that microbial CO_2_ bioconversion has developed through the interaction of multiple closely interconnected research domains.

**FIGURE 6:**
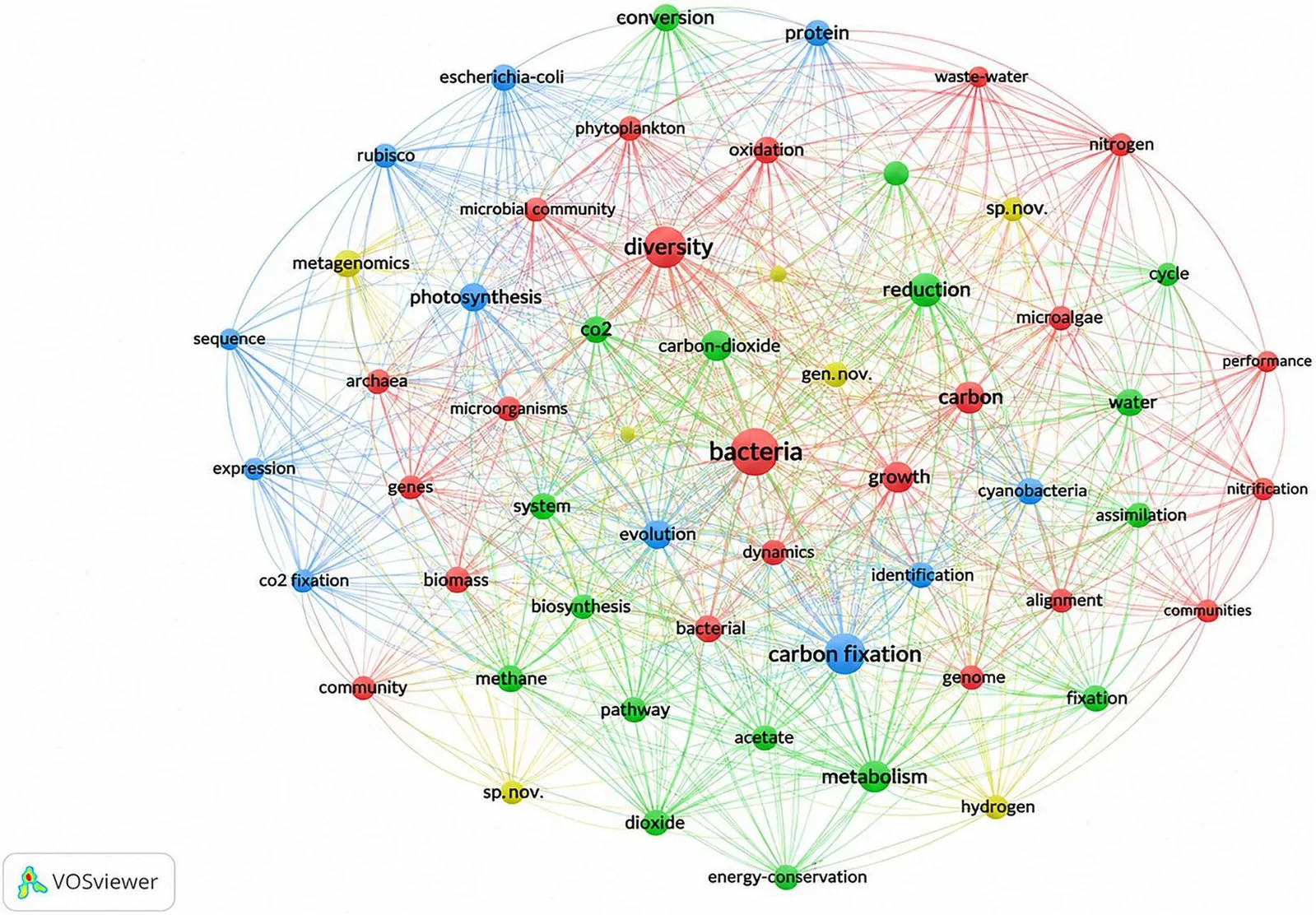
Keyword co-occurrence network and thematic structure in microbial CO_2_ bioconversion research. The network represents keywords meeting the minimum occurrence threshold of 20, with node size proportional to keyword occurrence, links representing co-occurrence relationships, and colors indicating thematic clusters identified by VOSviewer. The final network comprised 57 keywords distributed across four clusters, with 1,081 links and a total link strength of 1,084.00.

The red cluster is prominently structured around terms such as “bacteria,” “diversity,” “carbon,” “growth,” “genome,” “microbial community,” “oxidation,” “nitrogen,” and “waste-water.” This cluster primarily reflects the ecological and microbiological dimension of the literature, encompassing microbial diversity, community structure, microbial growth, genomic characterization, and interactions between carbon and other biogeochemical processes. Its strong connectivity with the remaining clusters indicates that microbial ecology continues to provide an important biological foundation for CO_2_-related biotechnology.

The green cluster contains highly connected terms including “CO_2_,” “carbon-dioxide,” “metabolism,” “methane,” “acetate,” “pathway,” “reduction,” “water,” “assimilation,” “fixation,” and “energy-conservation.” This thematic domain is particularly relevant to biological CO_2_ conversion because it connects carbon assimilation and fixation with microbial metabolism, energy conservation, reduced-carbon products, and metabolic pathways. The occurrence of methane and acetate within this cluster further illustrates the relationship between carbon fixation and microbial product formation.

The blue cluster is characterized by terms such as “carbon fixation,” “CO_2_ fixation,” “photosynthesis,” “rubisco,” “*Escherichia coli*,” “protein,” “sequence,” “expression,” “evolution,” and “identification.” These associations reflect a complementary molecular and mechanistic research axis involving carbon-fixation mechanisms, photosynthetic processes, protein function, genetic characterization, and molecular approaches. The presence of both classical carbon-fixation terminology and molecular biology-related keywords illustrates how fundamental understanding of carbon assimilation is increasingly connected with genetic and biochemical characterization.

Finally, the yellow cluster contains terms including “hydrogen,” “metagenomics,” “microorganisms,” “sp. nov.,” and related concepts positioned between the major biological clusters. Its network position suggests a bridging role connecting microbial discovery and characterization with energy-related and metabolic research. Particularly notable is the association of hydrogen with metabolism, acetate, carbon fixation, and CO_2_-related terminology, highlighting the close relationship between reducing-power availability, microbial energy metabolism, and biological carbon conversion.

Rather than representing isolated research areas, the four clusters are strongly interconnected, as demonstrated by the dense network of cross-cluster links. Terms such as “bacteria,” “CO_2_,” “carbon fixation,” “carbon-dioxide,” “metabolism,” “diversity,” and “growth” occupy prominent positions within the network and connect different thematic domains. This topology suggests that contemporary microbial CO_2_ bioconversion research increasingly integrates microbial ecology and diversity with carbon-fixation mechanisms, molecular characterization, metabolic pathways, energy conservation, and product formation.

Overall, the bibliometric analyses demonstrate that microbial CO_2_ bioconversion has developed into a rapidly expanding and increasingly interdisciplinary research field. The progression from 2,087 initially identified records to a rigorously selected corpus of 913 publications, together with the sustained temporal growth in scientific output, international and institutional concentration of research activity, broad disciplinary distribution, structured co-authorship network, and highly interconnected keyword landscape, reveals a field undergoing both scientific consolidation and technological diversification.

Importantly, the bibliometric landscape indicates that microbial CO_2_ bioconversion can no longer be viewed exclusively through the perspective of carbon fixation. Instead, the field increasingly connects microbial diversity, carbon assimilation, metabolic pathways, molecular characterization, energy conservation, and reduced-carbon product formation. This convergence provides the conceptual foundation for the following sections, which critically examine natural and engineered carbon-fixation pathways, microbial conversion platforms, and emerging strategies for biological CO_2_ valorization.

### 3.2 Carbon fixation pathways enabling microbial CO_2_ biomanufacturing

The increasing deployment of CCU technologies has shifted the focus of microbial carbon fixation research from describing metabolic diversity to identifying biological platforms capable of supporting efficient and scalable CO_2_ conversion. Although numerous natural and synthetic carbon fixation pathways have been described, their industrial relevance depends not only on their biochemical architecture but also on factors such as ATP efficiency, carbon conservation, product spectrum, scalability, and compatibility with industrial microbial hosts (Bierbaumer et al., 2023; Mustafa et al., 2026; Ravichandran et al., 2024). As illustrated in Figure 7, both natural and engineered pathways converge toward a limited number of central metabolic intermediates that serve as metabolic hubs for the biosynthesis of fuels, chemicals, polymers, proteins, and other value-added products.

**FIGURE 7:**
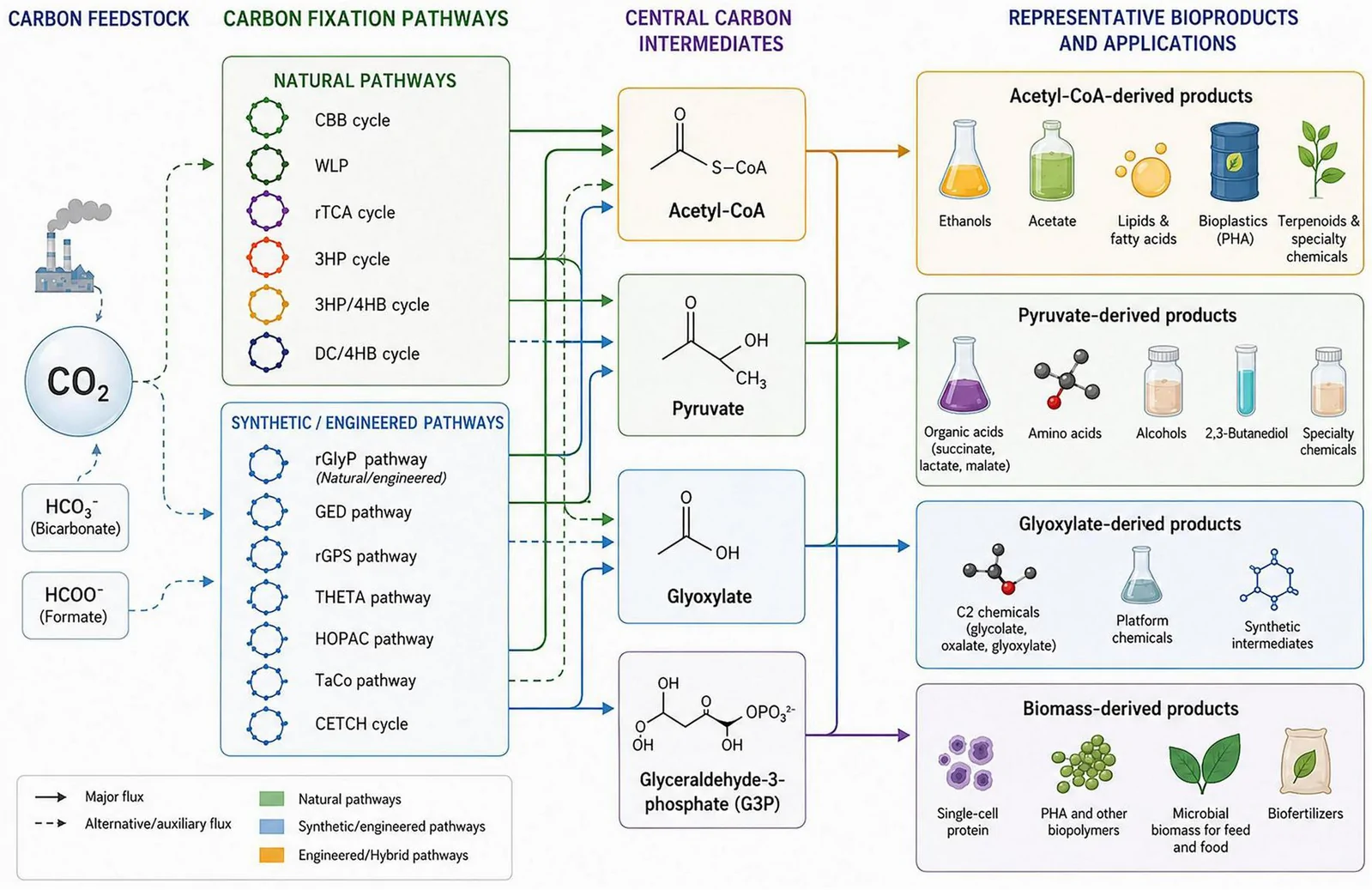
Natural and synthetic carbon fixation pathways convert CO_2_ into a limited set of central metabolic intermediates that serve as key metabolic hubs for microbial biomanufacturing. Natural pathways, including the Calvin–Benson–Bassham (CBB) cycle, Wood–Ljungdahl pathway (WLP), reductive glycine pathway (rGlyP), reductive tricarboxylic acid (rTCA) cycle, 3-hydroxypropionate (3HP) bicycle, 3HP/4HB cycle, and dicarboxylate/ 4-hydroxybutyrate (DC/4HB) cycle, assimilate carbon into intermediates such as acetyl-CoA, pyruvate, glyoxylate, and glyceraldehyde-3-phosphate (G3P). Synthetic pathways, including GED, reductive glycine–pyruvate synthesis (rGPS), THETA, HOPAC, TaCo, and CETCH, have been designed to improve carbon conservation, thermodynamic efficiency, and compatibility with industrial microbial hosts. These intermediates support the biosynthesis of a broad portfolio of CO_2_-derived products, including alcohols, organic acids, lipids, bioplastics, microbial proteins, and specialty chemicals.

From an industrial perspective, carbon fixation pathways should be viewed as metabolic platforms rather than merely biochemical routes for CO_2_ assimilation. Their intrinsic characteristics influence energy efficiency, carbon utilization, reactor configuration, electron donor requirements, metabolic flux distribution, and ultimately the techno-economic feasibility of CO_2_ biomanufacturing processes. As summarized in Table 1, most pathways channel assimilated carbon into a limited number of central metabolic intermediates, including acetyl-CoA, pyruvate, glyoxylate, and tricarboxylic acid (TCA) cycle intermediates, which largely define their suitability for producing distinct classes of bioproducts (Kumar and Eswari, 2023; Yang M. et al., 2026).

**TABLE 1:** Overview of natural and synthetic microbial CO_2_ fixation pathways.

CO_2_ fixation pathway	Type	Key enzyme(s)	Representative chassis	Main metabolic intermediate	ATP demand	O2 tolerance	Engineering accessibility	Industrial maturity
Calvin–Benson–Bassham cycle (CBB)	Natural	RuBisCO; phosphoribulokinase	*Cupriavidus necator*	Glyceraldehyde-3-phosphate (G3P)	High	High	High	High
Wood–Ljungdahl pathway (WLP)	Natural	Carbon monoxide dehydrogenase/acetyl-CoA synthase (CODH/ACS); formate dehydrogenase; THF-dependent enzymes	*Clostridium ljungdahlii*	Acetyl-CoA	Very low	Very low	Moderate	High
Reductive TCA cycle (rTCA)	Natural	ATP citrate lyase; pyruvate:ferredoxin oxidoreductase; 2-oxoglutarate:ferredoxin oxidoreductase	*Sulfurimonas denitrificans*	Acetyl-CoA / TCA intermediates	Low	Low	Low	Medium
3-Hydroxypropionate bicycle (3HP)	Natural	Acetyl-CoA carboxylase; propionyl-CoA carboxylase	*Chloroflexus aurantiacus*	Glyoxylate	High	Moderate	Low	Low
3HP/4HB cycle	Natural	Acetyl-CoA carboxylase; propionyl-CoA carboxylase; 4-hydroxybutyryl-CoA dehydratase	*Metallosphaera sedula*	Acetyl-CoA	Moderate	Moderate	Low	Low-Medium
Dicarboxylate/4-hydroxybutyrate cycle (DC/4HB)	Natural	Pyruvate:ferredoxin oxidoreductase; 4-hydroxybutyryl-CoA dehydratase	*Ignicoccus hospitalis*	Acetyl-CoA	Low	Low	Very low	Low
Reductive glycine pathway (rGlyP)	Natural/engineered	Formate dehydrogenase; glycine cleavage/synthase system; serine hydroxymethyltransferase	Engineered *Escherichia coli*	Pyruvate	Very low	High	High	Medium
GED pathway	Synthetic/engineered	6-Phosphogluconate dehydrogenase; Entner–Doudoroff pathway enzymes	Engineered *Escherichia coli*	Pyruvate / Glyceraldehyde-3-phosphate	Low	High	Moderate	Low
CETCH cycle	Synthetic	Enoyl-CoA carboxylase/reductase (ECR); CoA-dependent enzymes	Cell-free systems	Glyoxylate	Very low	High (cell-free)	Low	Low
THETA cycle	Synthetic	Modular carboxylases; CoA-dependent enzymes	Engineered microbial chassis	Acetyl-CoA	Very low	High*	Low	Very low
HOPAC cycle	Synthetic	Hydroxypropionate-related carboxylases; reductases	Engineered microbial chassis	Acetyl-CoA / Pyruvate	Low	High	Low	Very low
Reverse Glycine–Pyruvate Synthesis (rGPS) cycle	Synthetic/engineered	Formate assimilation enzymes; reverse glycine cleavage system; serine pathway enzymes	Engineered *Escherichia coli*	Pyruvate	Very low	High	Moderate	Low
Tartronyl-CoA (TaCo) pathway	Synthetic/emerging	Tartronyl-CoA synthase; CoA-dependent carboxylation enzymes	Engineered microbial chassis	Glyoxylate /Acetyl-CoA	Very low	High*	Low	Very low

*Predicted from pathway design. ATP demand refers to the relative energetic cost of carbon fixation. O2 tolerance indicates the compatibility of each pathway with aerobic cultivation. Engineering accessibility reflects the current feasibility of pathway implementation through metabolic engineering and synthetic biology. Industrial maturity was qualitatively classified according to the level of experimental and technological validation reported in the literature: Very low, pathway proposed computationally or demonstrated primarily at the enzymatic or cell-free proof-of-concept level, with no established microbial bioprocess validation; Low, experimental pathway demonstration under controlled laboratory conditions, with limited bioprocess validation and no substantial scale-up evidence; Medium, pathway implemented in native or engineered microbial hosts with laboratory-scale bioprocess validation and increasing evidence of process development, but limited industrial-scale deployment; and High, pathway implemented in established microbial production platforms with substantial bioprocess development, scale-up evidence, or industrially relevant application. These categories represent comparative industrial-maturity classes and should not be interpreted as formal numerical Technology Readiness Levels (TRLs). ATP demand, O2 tolerance, engineering accessibility, and industrial maturity were synthesized from the current literature. Information compiled and adapted from Claassens et al. (2020); Claassens (2021), Bierbaumer et al. (2023), Federici et al. (2023), Zhang et al. (2023), Della Valle et al. (2024), Tommasi (2024), Lee et al. (2025), Dronsella et al. (2025), O’Keeffe et al. (2025), Chen et al. (2026), and Zhao et al. (2026).

Importantly, no single carbon fixation pathway can currently be regarded as universally optimal. Instead, each represents a distinct evolutionary and engineering solution with specific advantages and trade-offs that depend on the microbial chassis, cultivation strategy, and target product. Accordingly, the industrial-maturity categories presented in Table 1 were assigned based on the extent of experimental validation, implementation in microbial hosts, bioprocess development, scale-up evidence, and industrially relevant application reported for each pathway. These categories provide a comparative indication of technological maturity rather than formal numerical Technology Readiness Level (TRL) assignments, which would require technology- and application-specific validation.

#### 3.2.1 Calvin–Benson–Bassham cycle

The Calvin–Benson–Bassham (CBB) cycle is the most widespread biological carbon fixation pathway and is predominantly associated with photoautotrophic organisms, including plants, algae, and cyanobacteria (Priyadharsini et al., 2022). In microbial biotechnology, however, the CBB cycle is also relevant in genetically tractable autotrophic chassis, including cyanobacteria and chemolithoautotrophic bacteria such as *Cupriavidus necator* H16, which has been explored for CO_2_-based biomanufacturing (Claassens et al., 2020; Dronsella et al., 2025). Although RuBisCO exhibits relatively low catalytic efficiency and the pathway requires substantial ATP investment, its robustness, compatibility with oxygenic or aerobic cultivation, and integration with central metabolism have supported its application in the production of polyhydroxyalkanoates, microbial proteins, biofuels, and specialty chemicals. From a biomanufacturing perspective, therefore, the principal strength of the CBB cycle lies not in its energetic efficiency but in the combination of its broad biological occurrence, physiological robustness, and implementation in experimentally tractable microbial hosts (Federici et al., 2023; Feng et al., 2023).

#### 3.2.2 Wood–Ljungdahl pathway

In contrast, the Wood–Ljungdahl pathway (WLP) is recognized as the most ATP-efficient natural carbon fixation pathway currently known. Acetogenic microorganisms, including *Acetobacterium woodii*, *Eubacterium limosum*, *Clostridium aceticum*, *C. ljungdahlii*, and *Moorella thermoacetica*, convert CO_2_ directly into acetyl-CoA through an energy-conserving reductive acetyl-CoA pathway supported by electron bifurcation and membrane-associated energy conservation systems (Kurt et al., 2023; Gasparrini et al., 2025; O’Keeffe et al., 2025). The direct formation of acetyl-CoA makes this pathway particularly suitable for producing acetate, ethanol, butyrate, fatty acids, wax esters, biodegradable polymers, and other reduced chemicals. However, its industrial implementation remains constrained by the strict anaerobic lifestyle of acetogens and the oxygen sensitivity of key metalloenzymes, particularly the CODH/ACS complex (Biester et al., 2022; Robles-Iglesias et al., 2023).

#### 3.2.3 Reductive glycine pathway

The reductive glycine pathway (rGlyP) has recently emerged as one of the most promising platforms for next-generation carbon biomanufacturing. Unlike conventional autotrophic pathways, rGlyP-based systems can assimilate formate produced by electrochemical CO_2_ reduction, thereby mitigating several gas–liquid mass transfer limitations associated with gaseous substrates. Engineered strains of *Escherichia coli*, *Pseudomonas putida*, and *Cupriavidus necator* have demonstrated the feasibility of coupling formate assimilation with microbial production, highlighting the potential of rGlyP as a key interface between renewable electricity and sustainable biomanufacturing (Bruinsma et al., 2023; Claassens et al., 2020; Li K. et al., 2025). Because carbon assimilation proceeds through pyruvate and other central metabolites, this pathway is particularly attractive for the biosynthesis of amino acids, organic acids, and specialty chemicals (Dronsella et al., 2025).

#### 3.2.4 rTCA, 3HP, 3HP/4HB, and DC/4HB pathways

Although the reductive tricarboxylic acid (rTCA), 3-hydroxypropionate (3HP), 3HP/4HB, and dicarboxylate/4-hydroxybutyrate (DC/4HB) pathways have not yet achieved widespread industrial implementation, they represent highly efficient natural carbon fixation strategies that continue to inspire the design of synthetic carbon assimilation pathways. Their remarkable biochemical diversity reflects the broad evolutionary landscape of microbial carbon fixation while providing valuable design principles for the development of next-generation engineered pathways (Claassens, 2021; Taha et al., 2025).

Despite their diversity, natural carbon fixation pathways evolved to maximize microbial fitness rather than industrial performance. Consequently, many remain constrained by high ATP requirements, slow carboxylation kinetics, oxygen sensitivity, and complex regulatory networks, limiting their direct application in industrial biotechnology (Bierbaumer et al., 2023; Zhang et al., 2023). These limitations have stimulated the rapid development of synthetic carbon fixation pathways aimed at improving thermodynamic efficiency, enhancing carbon conservation, reducing energy demand, and facilitating implementation in industrial microbial host (Li Z. et al., 2025; Mustafa et al., 2026).

#### 3.2.5 Synthetic pathways

Synthetic carbon fixation pathways represent a major advance in microbial biotechnology by enabling the rational redesign of carbon assimilation beyond the constraints of natural evolution. Pathways such as GED and reductive glycine–pyruvate synthesis (rGPS) were developed to efficiently generate pyruvate and glyceraldehyde-3-phosphate while minimizing ATP expenditure and facilitating integration with genetically tractable hosts such as *E. coli*. Similarly, the THETA, HOPAC, and TaCo pathways maximize carbon conservation during acetyl-CoA biosynthesis, providing promising platforms for producing alcohols, fatty acids, terpenoids, and biodegradable polymers (Satanowski et al., 2020; Dowaidar, 2024; Evans et al., 2024; McLean et al., 2023; Mustafa et al., 2026).

Among engineered pathways, the CETCH cycle remains one of the most influential proof-of-concept systems for artificial carbon fixation. Built around highly efficient enoyl-CoA carboxylase/reductase (ECR) reactions, this cell-free metabolic network demonstrated the feasibility of designing carbon fixation pathways with superior theoretical efficiency and carbon conservation compared with several naturally evolved pathways. Although its implementation in living microorganisms remains challenging, the CETCH cycle established an important conceptual framework for the rational engineering of next-generation carbon fixation pathways (Tommasi, 2024; Grim et al., 2026).

Despite their remarkable theoretical performance, most synthetic pathways remain at the laboratory stage owing to challenges related to enzyme compatibility, pathway regulation, metabolic burden, and stable expression in living cells. Consequently, their successful implementation depends not only on carbon fixation efficiency but also on host physiology, redox balance, oxygen tolerance, reactor configuration, substrate availability, and overall techno-economic performance (Ray et al., 2022; Barone et al., 2025; Puiggené et al., 2025).

Overall, natural and synthetic carbon fixation pathways should be viewed as complementary rather than competing solutions. While natural pathways provide the physiological robustness and technological maturity required for industrial deployment, synthetic pathways offer unprecedented opportunities to redesign carbon metabolism according to engineering principles. Future microbial CO_2_ biomanufacturing will likely rely on hybrid metabolic architectures that combine the robustness of natural carbon assimilation with the superior energetic and carbon-conservation potential of engineered pathways.

## 4 Emerging technologies for CO_2_ conversion

### 4.1 Bioelectrochemical systems and microbial electrosynthesis

Bioelectrochemical systems (BES) have emerged as a promising platform for coupling renewable electricity with microbial CO_2_ bioconversion, enabling the direct conversion of electrical energy into value-added carbon-based products. By integrating electrochemical reactions with the metabolic capabilities of electroactive microorganisms, BES enable the conversion of CO_2_ into fuels, chemicals, biomaterials, and other high-value products under mild operating conditions (Rojas-Flores et al., 2025; Nisa et al., 2026). Compared with conventional thermochemical CO_2_ conversion technologies, BES operate under ambient temperature and pressure, require lower energy inputs, and can be readily integrated with intermittent renewable electricity sources, making them attractive components of future low-carbon biomanufacturing systems when coupled with low-carbon electricity and appropriately sourced CO_2_ (Jatoi and Mubarak, 2026).

Among BES technologies, microbial electrosynthesis (MES) has emerged as one of the most extensively investigated platforms because it enables microorganisms to utilize electrons supplied from a cathode, either directly through extracellular electron transfer or indirectly via electrochemically generated hydrogen, to reduce CO_2_ into products such as acetate, alcohols, methane, medium-chain carboxylates, polyhydroxyalkanoates, and microbial biomass. This direct coupling of renewable electricity with microbial metabolism establishes MES as a promising interface between Power-to-X technologies and biological carbon utilization (Bakonyi et al., 2023; Suri et al., 2024; Berde et al., 2026; Lee et al., 2026).

Acetogenic bacteria are among the most extensively investigated biocatalysts for microbial electrosynthesis (MES) because they can couple CO_2_ fixation through the Wood–Ljungdahl pathway with reducing equivalents ultimately derived from the cathode. *Sporomusa ovata* has emerged as one of the most prominent model electrotrophs for electroacetogenesis, while *Acetobacterium woodii* and *Clostridium ljungdahlii* have also been investigated as CO_2_-converting biocatalysts. However, substantial organism-dependent differences in performance have been reported. For example, *S. ovata* reached an acetate production rate of 51.1 g m^–2^ d^–1^, compared with 12.8 g m^–2^ d^–1^ for *A. woodii* and 0.14 g m^–2^ d^–1^ for *C. ljungdahlii*, while current densities of up to 22 A m^–2^ have been reported for *S. ovata* (Madjarov et al., 2022). These differences emphasize that biocatalyst selection, electron-uptake capacity, product formation rate, and selectivity remain critical determinants of MES performance.

Electron delivery in MES can occur through mechanistically distinct routes with different reactor and energy requirements. In direct electron transfer (DET), microorganisms associated with the cathode receive reducing equivalents through electrode–cell interactions, making biofilm formation, cathode properties, and interfacial electron-transfer kinetics important determinants of process performance. Recent electrode-engineering strategies have demonstrated that improving the biocathode–microorganism interface can enhance electron transfer and CO_2_ conversion in MES (Noori et al., 2025). In contrast, H_2_-mediated electron transfer relies on cathodic H_2_ evolution, with the generated H_2_ serving as a diffusible reducing equivalent for hydrogenotrophic microorganisms. Although this mechanism reduces dependence on direct electrode–cell contact, it introduces additional requirements related to H_2_ evolution, cathodic overpotential, and gas–liquid mass. Consequently, DET and H_2_ -mediated electron transfer are not energetically or operationally equivalent, and these differences must be considered when optimizing electrode materials, applied potential, reactor configuration, and overall process efficiency (Bakonyi et al., 2023; Omidi et al., 2024).

Despite these advances, MES still faces important biological and electrochemical limitations that restrict its transition toward industrially relevant operation. Low product titers and production rates, insufficient current uptake, variable coulombic efficiencies, limited product selectivity, and challenging scale-up remain major bottlenecks. These limitations are closely linked to the mechanism by which reducing equivalents reach the microbial catalyst. In *C. ljungdahlii*, cathodically generated H_2_ has been demonstrated to act as a dominant electron mediator, and increased H_2_ availability enhanced growth, metabolic activity, acetate titers, and production rates, reaching 6.06 g L^–1^ acetate and 0.11 g L^–1^ d^–1^ (Boto et al., 2023). Consequently, improving MES requires coordinated optimization of microbial physiology, electron-transfer mechanisms, cathode architecture, current density, mass transfer, coulombic efficiency, product selectivity, and reactor configuration rather than optimization of the electrode or microorganism independently (Madjarov et al., 2022; Boto et al., 2023).

Despite substantial technological progress, BES and MES remain at relatively low technology readiness levels (TRLs). Current limitations include inefficient extracellular electron transfer, low current densities, cathode overpotentials, biofilm instability, limited product selectivity, mass-transfer constraints, and challenges associated with reactor scale-up (Ieropoulos et al., 2024). Recent advances in electrode engineering, conductive nanomaterials, reactor design, systems biology, and metabolic engineering have significantly improved electron transfer efficiency and redirected carbon flux toward target products Nevertheless, further improvements in reactor productivity, energy efficiency, long-term operational stability, and techno-economic performance remain essential before BES can achieve widespread industrial deployment for large-scale CO_2_ valorization. The convergence of renewable electricity, electrochemical engineering, synthetic biology, and advanced reactor design is expected to accelerate the development of next-generation BES platforms. As electrode materials, reactor configurations, and engineered microbial chassis continue to evolve, bioelectrochemical systems are likely to become an increasingly important component of integrated CCU strategies, supporting sustainable fuel production, carbon recycling, and low-carbon biomanufacturing (Li et al., 2023; Omidi et al., 2024; Rojas-Flores et al., 2025).

Beyond conventional MES, hybrid electrochemical–biological systems provide an alternative strategy for coupling CO_2_ reduction with heterotrophic bioconversion. Hann et al. (2022) demonstrated a tandem electrochemical process converting CO_2_ into acetate with 57% carbon selectivity, generating an acetate-rich stream that supported heterotrophic cultivation of *Chlamydomonas reinhardtii* and *Saccharomyces cerevisiae*. In *S. cerevisiae*, the electrolysis-derived acetate supported a biomass yield of 0.19 g g^–1^ acetate and a ninefold increase in OD_600_, illustrating the potential of electrochemically generated intermediates to bridge CO_2_ reduction and microbial biomanufacturing.

More broadly, MES should be considered within the wider portfolio of CO_2_-conversion technologies. Thermal, electrochemical, photochemical, and biochemical routes differ in their energy inputs, catalytic requirements, operating conditions, selectivity, and scale-up constraints. Within this landscape, MES occupies a distinctive position by integrating electrochemical energy delivery with microbial catalysis under comparatively mild operating conditions, although its competitiveness remains dependent on improvements in electron-transfer efficiency, reactor productivity, energy efficiency, and scalability (Meshkani et al., 2027).

### 4.2 Synthetic microbial consortia

Although considerable progress has been achieved in engineering individual microorganisms, the metabolic burden associated with simultaneously optimizing carbon fixation, precursor generation, redox balance, and product synthesis can constrain the performance of single-strain platforms. Synthetic microbial consortia provide an alternative strategy by distributing metabolic functions among complementary microorganisms, enabling division of labor, modular pathway organization, broader substrate and product spectra, and potentially greater process robustness (Li D. et al., 2025; Mustafa et al., 2026; Sauer and Marx, 2023). In CO_2_-based systems, this concept can be implemented by coupling an autotrophic or photoautotrophic carbon-fixing organism with a heterotrophic production host, whereby fixed carbon is transferred as an intermediate metabolite and subsequently converted into a target product. Such metabolic compartmentalization can reduce the metabolic burden imposed on individual strains while allowing each member to be optimized for a more specialized function (Gao et al., 2022; Sauer and Marx, 2023).

Light-driven synthetic consortia provide a representative example of this architecture. Engineered *Synechococcus elongatus* can fix CO_2_ using light energy and export sucrose, which subsequently supports product formation by heterotrophic partners. For instance, coupling *S. elongatus* with *Pseudomonas putida* enabled PHA production directly from CO_2_, reaching a maximum titer of 156 mg L^–1^ and productivity of 23.8 mg L^–1^ d^–1^. The modularity of this strategy has also enabled *S. elongatus* to be combined with different heterotrophic hosts, including *Azotobacter vinelandii*, *Escherichia coli*, and oleaginous yeasts, for the production of PHB, 3-hydroxypropionic acid, and microbial lipids, illustrating how a common CO_2_-fixing module can be coupled to different downstream production modules (Gao et al., 2022). Similarly, Sauer and Marx (2023) highlighted a *S. elongatus–P. putida* consortium for PHA production from CO_2_ and light, reporting a PHA titer of 393 mg L^–1^ and a production rate of 42.1 mg L^–1^ d^–1^, further demonstrating the potential of division-of-labor strategies for direct carbon valorization.

Despite these advantages, the increased metabolic flexibility of synthetic consortia is accompanied by additional engineering constraints. Differences in growth rates, nutrient requirements, oxygen demand, temperature and pH optima can destabilize population ratios, while imbalanced cross-feeding may cause intermediate accumulation, carbon loss, or competitive overgrowth of one partner. In light-driven systems, differences in osmotic requirements and the formation of reactive oxygen species can further compromise the viability of heterotrophic partners (Gao et al., 2022; Li et al., 2026). Consequently, successful scale-up requires not only optimization of individual strains but also control of population dynamics, metabolite exchange, spatial organization, and interspecies interactions. Strategies including metabolic modeling, controlled nutrient dependencies, spatial compartmentalization, immobilization, and dynamic regulation of microbial populations are therefore increasingly being explored to improve consortium stability and productivity (Sauer and Marx, 2023; Wang Z. et al., 2025). These advances position synthetic microbial consortia as modular platforms for CO_2_ biomanufacturing, while long-term stability, productivity, process control, and scale-up remain key barriers to industrial implementation (Yadav et al., 2025).

### 4.3 Next-generation CO_2_ cell factories

Unlike synthetic microbial consortia, which distribute metabolic functions among multiple species, next-generation microbial cell factories seek to integrate carbon fixation, energy conservation, precursor supply, and product synthesis within a single engineered chassis (Liu et al., 2025). Advances in metabolic engineering and synthetic biology have increasingly enabled the redesign of native CO_2_ fixing microorganisms through genome engineering, cofactor balancing, carbon-flux redistribution, pathway introduction, and synthetic regulatory systems. Rather than simply increasing CO_2_ assimilation, these strategies aim to coordinate carbon fixation with product-forming pathways while minimizing carbon losses to biomass and competing metabolic routes, thereby improving product specificity, yield, and metabolic robustness (San Román et al., 2025; Yang Y. et al., 2026).

*Cupriavidus necator* H16 represents a prominent model for this next generation of CO_2_ cell factories because of its metabolic flexibility and capacity for chemolithoautotrophic growth using H_2_ and CO_2_, with carbon assimilation occurring through the CBB cycle. Its native capacity for polyhydroxybutyrate (PHB) accumulation has provided a foundation for industrial biopolymer production, while metabolic engineering has substantially expanded its product portfolio toward non-native fuels, chemicals, bioactive compounds, and other value-added products. Moreover, the ability of *C. necator* to utilize H_2_/CO_2_, formate, and organic substrates enables autotrophic, formatotrophic, heterotrophic, and mixotrophic process configurations, making this organism a versatile chassis for integrating metabolic engineering with gas fermentation and emerging electromicrobial production strategies (Tang et al., 2023; Bansal et al., 2026).

However, constructing an effective CO_2_ cell factory requires more than introducing product-forming pathways. Autotrophic production remains constrained by the energetic and reducing-power requirements of carbon fixation, gas–liquid mass transfer, availability of H_2_ or alternative electron donors, competition between biomass formation and product synthesis, and insufficient metabolic flux toward non-native products. These constraints can result in lower productivities or yields even when CO_2_ assimilation is successfully established. Consequently, next-generation engineering increasingly combines CRISPR-based genome modification, adaptive laboratory evolution, systems-level omics, genome-scale metabolic modeling, and dynamic metabolic regulation to coordinate carbon fixation, energy metabolism, and product synthesis (Tang et al., 2023; Kösters and Marienhagen, 2025; Bansal et al., 2026). Future development should therefore focus not only on expanding the accessible product portfolio but also on achieving industrially relevant carbon efficiency, productivity, metabolic stability, and process robustness under autotrophic operating conditions (Zeng et al., 2022; Mustafa et al., 2026).

### 4.4 Artificial intelligence for carbon biomanufacturing

Artificial intelligence (AI) and machine-learning (ML) approaches are increasingly being integrated into microbial strain engineering and bioprocess development, particularly within Design–Build–Test–Learn (DBTL) workflows. In microbial CO_2_ biomanufacturing, their potential extends beyond general data analysis to the identification of metabolic bottlenecks, prediction of genotype–phenotype relationships, prioritization of engineering targets, and optimization of carbon and reducing-equivalent allocation. When combined with genome-scale metabolic models (GEMs) and multi-omics datasets, data-driven approaches can reduce the experimental search space by prioritizing gene deletions, overexpression targets, pathway modifications, and cultivation conditions before experimental validation (Shah et al., 2025; Paul et al., 2026).

A second emerging application concerns the design and optimization of enzymes and carbon-fixation pathways. Protein structure prediction, protein language models, and machine-learning-assisted enzyme engineering provide opportunities to evaluate sequence–structure–function relationships and identify variants with altered catalytic properties, substrate specificity, stability, or expression. Such approaches are particularly relevant to CO_2_ bioconversion because the performance of natural and synthetic carbon-fixation pathways is frequently constrained by slow carboxylation kinetics, enzyme compatibility, cofactor requirements, and intracellular regulation. AI-assisted enzyme and pathway design may therefore complement conventional metabolic engineering by enabling more systematic exploration of large sequence and pathway-design spaces (Panagiotidou and Theodosiou, 2026).

Artificial intelligence-assisted approaches can also operate at the bioprocess level. Integration of online process measurements with mechanistic models, high-throughput experimentation, automated cultivation platforms, and data-driven control strategies can facilitate optimization of variables such as gas composition, mass transfer, nutrient availability, pH, and biomass productivity. Coupling these tools with DBTL workflows creates a closed-loop framework in which experimental data continuously inform strain and process redesign, potentially accelerating optimization of CO_2_-utilizing microbial platforms (Agoni et al., 2025; Wang et al., 2026).

Nevertheless, the application of AI specifically to microbial CO_2_ biomanufacturing remains considerably less mature than its broader use in protein engineering and industrial biotechnology. Model performance depends strongly on the quantity, quality, standardization, and biological diversity of training data, while predictions obtained from well-characterized model organisms may not readily transfer to autotrophic or non-model CO_2_-fixing microorganisms. Moreover, many AI-enabled applications proposed for CO_2_ biomanufacturing remain prospective or have been demonstrated primarily in adjacent areas of biotechnology rather than directly validated in CO_2_-based production systems. Predicted metabolic interventions, enzyme variants, and process-control strategies therefore require experimental validation under physiologically and industrially relevant conditions. AI should consequently be regarded as a complementary decision-support and design tool rather than a substitute for mechanistic metabolic understanding and experimental validation. Its greatest contribution to CO_2_ biomanufacturing may arise from integrating computational prediction, systems biology, automated experimentation, and bioprocess engineering within iterative DBTL cycles (Kumar et al., 2026; Nneoma et al., 2025).

## 5 Product portfolio enabled by microbial CO_2_ bioconversion

Microbial CO_2_ bioconversion enables the production of fuels, chemicals, materials, and food ingredients through natural and engineered carbon-assimilation pathways. Advances in metabolic engineering, synthetic biology, gas fermentation, and bioelectrochemical systems have expanded the accessible product portfolio and strengthened the potential of microbial platforms for sustainable carbon utilization (Zhao et al., 2026). The following sections examine the major product classes, emphasizing representative microbial systems, technological advances, industrial applications, and maturity levels, while quantitative performance data are summarized in Table 2.

**TABLE 2:** Representative products obtained through microbial CO_2_ bioconversion and bioelectrochemical platforms.

Product category	Product	Representative microorganism/system	CO_2_ conversion platform	Carbon source/electron donor	Representative performance (metric type)	Main application	References
Alcohols	Ethanol	*Clostridium autoethanogenum* DSM 23693	Continuous syngas fermentation (chemostat)	CO/ CO_2_/ H_2_	Titer: 4.5 g L^–1^; specific productivity (qEtOH): ≈ 8 mmol gDCW^–1^ h^–1^	Renewable fuel; platform chemical	de Lima et al., 2022
	Ethanol	*Clostridium autoethanogenum* DSM 19630	Autotrophic gas fermentation (chemostat)	CO_2_/H_2_ +CO	Titer: 2.36–9.69 g L^–1^; carbon distribution: 54–66 C-mol%	Renewable fuel; platform chemical	Heffernan et al., 2020
	Ethanol	*Clostridium carboxidivorans* DSM 15243	Continuous autotrophic gas fermentation	C1 waste gas	Titer: 1.82 g L^–1^	Renewable fuel; platform chemical	Pisacane et al., 2024
	Isopropanol	Mixed culture dominated by *Acetobacterium*	MES	CO_2_/ electricity	Areal productivity: 1.17 ± 0.34 g m^–2^ d^–1^	Solvent, chemical intermediate	Arends et al., 2017
	Butanol	*Clostridium carboxidivorans* DSM 15243	Continuous autotrophic gas fermentation	C1 waste gas	Titer: 0.33 g L^–1^ (pH 5.75)	Advanced biofuel; chemical intermediate	Pisacane et al., 2024
	2,3-Butanediol	*Clostridium autoethanogenum* DSM 23693	Continuous syngas fermentation (chemostat)	CO/ CO_2_/ H_2_	Titer: 0.8 g L^–1^; increased specific productivity at higher growth rates	Industrial chemical; polymer precursor	de Lima et al., 2022
Organic acids	Acetate	*Acetobacterium woodii*	High-pressure autotrophic gas fermentation	CO_2_/ H_2_	Titer: 10.2 g L^–1^; specific productivity 22.06 g g^–1^ d^–1^	Platform chemical; precursor for chain elongation and C2–C6 chemicals	Tarraran et al., 2023
	Acetate	Mixed anaerobic culture	MES	CO_2_/electricity	Areal productivity: 685 ± 30 g m^–2^ d^–1^	Platform chemical, power-to-chemicals	Jourdin et al., 2015
	Formate	Engineered *Acetobacterium woodii*	High-pressure autotrophic gas fermentation	CO_2_/ H_2_	Titer: 5.85 g L^–1^; volumetric productivity: 5.12 g L^–1^ d^–1^	C1 platform molecule; hydrogen storage; microbial feedstock	Tarraran et al., 2023
	Formate	*Clostridium ljungdahlii*	High-pressure anaerobic bioreactor	CO_2_/ H_2_	Titer: 3.3 g L^–1^	C1 platform molecule, microbial feedstock	Oswald et al., 2018
	Lactate	Recombinant *Acetobacterium woodii* expressing *Leuconostoc mesenteroides* LDH	Autotrophic gas fermentation (Wood–Ljungdahl pathway)	CO_2_/ H_2_	Concentration: 18.8 mM	PLA precursor; biodegradable plastics; chemical industry	Moon et al., 2023
	*n*-Butyrate	Mixed microbial biofilm	Continuous bioelectrochemical chain elongation	CO_2_/ electricity	Volumetric productivity: 3.3 ± 0.2 g L^–1^ d^–1^	Platform chemical; precursor for fuels and chain elongation	Jourdin et al., 2019
	*n*-Caproate	Mixed microbial biofilm	Continuous bioelectrochemical chain elongation	CO_2_/ electricity	Volumetric productivity: 2.0 ± 0.1 g L^–1^ d^–1^	Medium-chain carboxylate; fuel and chemical precursor	Jourdin et al., 2019
	3-Hydroxypropionate	Engineered *Cupriavidus necator* H16 (Δ3 ΔgdhA1 ΔhpdH)	Autotrophic gas fermentation	CO_2_/ H_2_	Concentration: 0.5 mM	Acrylic acid precursor; biodegradable polymers; specialty chemicals	Salinas et al., 2022
	Succinate	*Citrobacter amalonaticus*-related isolate	Anaerobic heterotrophic cultivation with CO_2_ fixation	Sucrose + CO_2_/ H_2_	Volumetric productivity: 12.07 g L^–1^ d^–1^	Biopolymers, food and pharmaceutical sectors	Amulya and Mohan, 2019
Lipids and biodiesel	FAMEs	*Bacillus cereus* SM1	Chemolithotrophic CO_2_ conversion	CO_2_/ Fe^2+^	Biodiesel yield: 86.5%	Biodiesel	Mishra et al., 2016
	Long-chain fatty alcohols	*Halomonas stevensii* KP163920	Semi-continuous aerobic CO_2_ bioconversion	CO_2_/ Na_2_S_2_O3	Biomass-specific product content: 606.72 mg g^–1^ biomass	Surfactants, detergents, lubricants, biofuels	Mishra et al., 2018
	FAMEs	Mixed microbial culture (activated sludge)	Chemolithotrophic CO_2_ conversion	CO_2_/ Fe^2+^	Biodiesel yield: 91.55%	Renewable transportation fuels	Mishra et al., 2019
	FAMEs	*Enterobacter cloacae* PP1	Chemolithotrophic CO_2_ conversion	CO_2_/ Fe^2+^	Biodiesel yield: 77.49%	Renewable transportation fuels	Mishra et al., 2019
	FAMEs	*Pseudomonas putida* SM2	Chemolithotrophic CO_2_ conversion	CO_2_/ Fe^2+^	Biodiesel yield: 38.69%	Renewable transportation fuels	Mishra et al., 2019
	Microbial lipids and hydrocarbons (C13–C24)	*Serratia* sp. ISTD04	Chemolithotrophic cultivation with carbon-concentrating mechanism (CCM)	NaHCO_3_	Lipid concentration: 72.19 mg/100 mL; hydrocarbon concentration: 42.6 mg/100 mL	Biodiesel precursor; jet fuel-range hydrocarbons	Kumar et al., 2017
	FAMEs	*Serratia* sp. ISTD04 + immobilized *Pseudomonas* sp. ISTPL3 lipase	Enzymatic transesterification	CO_2_-derived lipids	Purity: 83.5%; conversion: 97.41%	Biodiesel	Kumar and Thakur, 2020
Bioplastics	PHB	*Cupriavidus necator* H16 (CbbR-RegA recombinant strain)	Lithoautotrophic gas fermentation (Calvin–Benson–Bassham cycle)	CO_2_/ H_2_	Titer:1.2 g L^–1^; polymer content: 58% CDW	Biodegradable plastics	Kim et al., 2022
	PHB	*Cupriavidus necator* H16 (*rpoN*-overexpressing strain)	Chemoautotrophic cultivation	CO_2_/ H_2_	Relative production: +77.5%	Biodegradable plastics; biomedical materials	Tang et al., 2022
	PHB	*Cupriavidus necator* H16 (*phaP1*-overexpressing strain)	Chemoautotrophic cultivation	CO_2_/ H_2_	Relative production: +49.8%	Biodegradable plastics; biomedical materials	Tang et al., 2022
	PHA	*Cupriavidus* sp. CY-1	Autotrophic gas fermentation	CO_2_/ H_2_/ O_2_	Titer: 11.87 g L^–1^; polymer content: 90.6% CDW	Biodegradable plastics	Chang et al., 2025
	PHBV	*Cupriavidus* sp. CY-1	Two-stage autotrophic fermentation + valeric acid	CO_2_/ H_2_ + valeric acid	Titer: 1.47 g L^–1^	Medical devices, agricultural films	Chang et al., 2025
Proteins and food ingredients	SCP	*Cupriavidus necator* 335 (DSM 531)	Aerobic autotrophic (“Knallgas“) fermentation	CO_2_/ H_2_/ O_2_	Crude protein content: 83% (NH_4_^+^); volumetric biomass productivity: 60 mg CDW L^–1^ h^–1^	Sustainable protein for food and feed	Yang et al., 2021
	SCP	*Cupriavidus necator*	Autotrophic gas fermentation	CO_2_/ H_2_/ O_2_	Protein content: 69% DW	Food and feed	Ismail et al., 2024
	SCP	*Cupriavidus necator* H16	Passive gas-fed autotrophic cultivation	CO_2_/ H_2_	Volumetric biomass productivity: 58 mg L^–1^ h^–1^	Food production in resource-limited regions	Li B. et al., 2025
	SCP	*Cupriavidus necator* H16	Pilot-scale pressurized gas fermentation	CO_2_/ H_2_/ O_2_	Biomass titer: 90 g L^–1^ CDW; crude protein content: 80%	Industrial SCP production	Novak et al., 2025
	SCP	*Clostridium ljungdahlii*–*Saccharomyces cerevisiae*	Two-stage sequential cultivation	CO_2_/ H_2_ -derived acetate	Volumetric productivity: 1.3 g L^–1^ h^–1^	Alternative protein	Molitor et al., 2019
Specialty biomolecules	EPS	*Serratia* sp. ISTD04	Calvin–Benson–Bassham-based chemolithotrophic cultivation	NaHCO_3_ + CO_2_ (glucose as inducer)	Titer: 4.57 ± 0.27 g L^–1^	Bioflocculants, dye removal, food and biomedical biopolymers	Kumar et al., 2019
	Wax esters	*Acinetobacter baylyi*	MES	CO_2_/ electricity	Titer: 0.02 g L^–1^	Lubricants, cosmetics, specialty chemicals	Lehtinen et al., 2017
	Biosurfactant	*Bacillus* sp. ISTS2	Chemolithotrophic CO_2_ sequestration via CCM (carbonic anhydrase–RuBisCO)	5% CO_2_ /100 mM NaHCO_3_	Surface tension: 33 mN m^–1^ (CO_2_) and 38 mN m^–1^ (NaHCO_3_); emulsification index (E24): 53% and 41%, respectively	Environmental remediation, bioemulsifiers, sustainable surfactants	Sundaram and Thakur, 2015

The studies presented are representative examples of microbial and bioelectrochemical CO_2_ bioconversion and do not constitute an exhaustive survey of all reported products. Representative performance metrics are reported according to the original studies and include product titer or concentration, volumetric, specific or areal productivity, product yield, carbon distribution, biomass-specific product content, polymer or protein content, conversion, and other process-specific indicators. Because these metrics were obtained under different microbial systems, cultivation conditions, carbon and electron sources, reactor configurations, and analytical bases, they should not be interpreted as direct quantitative rankings across microbial platforms. Comparisons should primarily be made within equivalent metric classes and broadly comparable cultivation configurations. Carbon source/electron donor refers to the principal substrates supplied during cultivation. CDW, cell dry weight; FAMEs, fatty acid methyl esters; PHA, polyhydroxyalkanoates; PHB, poly(3-hydroxybutyrate); PHBV, poly(3-hydroxybutyrate-co-3-hydroxyvalerate); SCP, single-cell protein; EPS, exopolysaccharide; MES, microbial electrosynthesis.

The heterogeneous performance metrics reported in Table 2 should be interpreted according to their respective analytical and process bases rather than as directly interchangeable measures of technological performance. Product titer reflects the final product accumulation achieved under a given cultivation condition, whereas productivity describes the rate of product formation and is therefore more directly related to reactor throughput. Yield and carbon-efficiency metrics provide complementary information on resource utilization and the fraction of supplied carbon effectively directed toward the target product. Consequently, high performance in one metric does not necessarily imply superior overall process performance; for example, a high product titer may be associated with extended cultivation times, while high productivity may coexist with inefficient carbon utilization. Meaningful comparisons should therefore prioritize equivalent metrics obtained under broadly comparable cultivation and reactor configurations.

These distinctions are particularly relevant when evaluating the sustainability of microbial CO_2_ bioconversion. High titer, productivity, or carbon incorporation alone does not establish environmental superiority, because the overall carbon and energy balance also depends on the origin and conditioning of CO_2_, the source of reducing power or H_2_, electricity carbon intensity, reactor energy requirements, downstream separation and purification, and product lifetime or subsequent carbon release. Accordingly, microbial CO_2_-derived products should be evaluated using integrated performance indicators together with life-cycle, energy, and techno-economic criteria. Recent sustainability assessments of CO_2_-derived materials similarly emphasize the importance of low-carbon energy inputs, efficient resource utilization, and carbon circularity, while cautioning that circular carbon use does not intrinsically guarantee environmental sustainability (Ramirez-Corredores, 2024).

### 5.1 Alcohols

Alcohols represent a major product class accessible through microbial CO_2_ and C1-gas bioconversion owing to their applications as renewable fuels, industrial solvents, and chemical building blocks. Gas fermentation currently provides the most mature production route, whereas microbial electrosynthesis and engineered metabolic pathways are expanding the spectrum toward higher-value and more reduced alcohols (Kaliyaperumal et al., 2023; Li et al., 2024).

#### 5.1.1 Ethanol

Ethanol is a renewable fuel and versatile platform chemical used in the manufacture of ethylene, sustainable aviation fuel, solvents, and other bio-based products. It also represents the most technologically mature product of industrial gas fermentation, with acetogenic bactéria, particularly *Clostridium autoethanogenum*, serving as the principal microbial chassis (Dagle et al., 2020; Köpke and Simpson, 2020; Li et al., 2024).

Recent studies have clarified how gas composition, microbial physiology, and reactor operation regulate ethanol formation. Heffernan et al. (2020) showed that low-level CO supplementation redirected carbon flux toward solventogenesis during CO_2_/H_2_ fermentation. de Lima et al. (2022) subsequently demonstrated that increased specific growth rates enhanced ethanol-specific productivity during continuous syngas fermentation. Using *Clostridium carboxidivorans*, Pisacane et al. (2024) further showed that pH strongly influences solvent distribution, with uncontrolled acidification favoring ethanol and controlled conditions promoting longer-chain alcohols. Collectively, these studies demonstrate that ethanol production depends on coordinated optimization of gas composition, cellular physiology, and reactor conditions.

#### 5.1.2 Butanol

Butanol is an advanced biofuel and industrial solvent with higher energy density, lower hygroscopicity, and greater compatibility with existing fuel infrastructure than ethanol (Petrov et al., 2021; Obergruber et al., 2021). Its applications extend to the chemical, pharmaceutical, coatings, and polymer industries. Autotrophic butanol production remains less mature than ethanol production and relies primarily on solventogenic acetogens such as *Clostridium carboxidivorans*. Pisacane et al. (2024) demonstrated that pH regulation during continuous CO_2_ fermentation altered acid-to-solvent conversion and favored the formation of longer-chain alcohols. This result highlights reactor operation as a critical determinant of butanol selectivity and indicates that process control may be as important as strain performance for expanding C4 alcohol production from C1 gases.

#### 5.1.3 Isopropanol

IIsopropanol is a high-value solvent used in pharmaceutical, cosmetic, electronic, and chemical manufacturing. In contrast to ethanol, its production from CO_2_ remains at an early technological stage and has been demonstrated primarily through microbial electrosynthesis and engineered gas-fermentation platforms (Li et al., 2024). Arends et al. (2017) provided an early proof-of-concept by demonstrating continuous isopropanol production from CO_2_ using an electroactive mixed microbial community. The study showed that cathodic electron flow could support products beyond acetate and other simple carboxylates, thereby extending the scope of microbial electrosynthesis toward more reduced and commercially valuable molecules. Its main contribution lies less in the absolute productivity than in demonstrating the feasibility of coupling renewable electricity directly to biological solvent production.

#### 5.1.4 2 ,3-Butanediol

Although classified as a diol rather than a monohydric alcohol, 2,3-butanediol is an important C4 platform chemical and precursor of methyl ethyl ketone, synthetic rubber, solvents, and polymer intermediates (Ji et al., 2011). Its production illustrates the ability of acetogenic platforms to generate chemicals beyond conventional alcohol fuels. de Lima et al. (2022) demonstrated that increasing the specific growth rate of *C. autoethanogenum* enhanced carbon allocation toward 2,3-butanediol during continuous syngas fermentation. This finding identifies microbial growth physiology as a key control point for diversifying the reduced-product portfolio of gas fermentation.

Collectively, these studies show that alcohol production from C1 gases is governed by interactions among microbial chassis, feed-gas composition, redox balance, growth physiology, and reactor operation. Ethanol currently defines the commercial benchmark, whereas butanol, isopropanol, and 2,3-butanediol illustrate the progressive expansion of microbial CO_2_ conversion toward higher-value products. Quantitative comparisons among these systems are presented in Table 2.

### 5.2 Organic acids

Organic acids constitute one of the most diverse and industrially relevant product classes derived from microbial CO_2_ bioconversion. These molecules function as versatile platform chemicals and metabolic intermediates for the manufacture of polymers, solvents, pharmaceuticals, food additives, biodegradable materials, and numerous specialty chemicals. Recent advances in gas fermentation, microbial electrosynthesis, metabolic engineering, and synthetic biology have considerably expanded the portfolio of organic acids accessible from CO_2_, reinforcing their strategic role in CCU, circular carbon management, and sustainable biomanufacturing (Li et al., 2024; Sakarika et al., 2024; Jofre et al., 2026). The quantitative outcomes of this study are presented in Table 2.

#### 5.2.1 Acetate

Acetate is the primary product of autotrophic acetogenesis and one of the most important platform chemicals derived from microbial CO_2_ bioconversion. Beyond its direct industrial applications, acetate serves as a central intermediate for microbial chain elongation, enabling the biosynthesis of medium-chain carboxylates, alcohols, and other value-added chemicals. Owing to its pivotal role in acetogenic metabolism, acetate remains the major product of autotrophic gas fermentation and an essential intermediate for downstream microbial upgrading using acetogenic bacteria (Bae et al., 2024; Wu et al., 2024; Khalid et al., 2024).

Recent studies have demonstrated that both microbial engineering and reactor intensification can substantially improve autotrophic acetate production. Using an engineered *Acetobacterium woodii*, Tarraran et al. (2023) showed that pressurized CO_2_/H_2_ fermentation enhanced gas availability and carbon fixation efficiency while simultaneously redirecting carbon metabolism toward desired products. The work illustrates how coupling metabolic engineering with high-pressure bioreactor operation can overcome gas–liquid mass transfer limitations and improve the industrial performance of acetogenic platforms.

Microbial electrosynthesis has further expanded acetate production by coupling renewable electricity with biological CO_2_ reduction. Using a three-dimensional biocathode colonized by a mixed anaerobic microbial community, Jourdin et al. (2015) demonstrated exceptionally efficient conversion of cathodic electrons into acetate. This study established microbial electrosynthesis as one of the first high-performance bioelectrochemical platforms for power-to-chemicals applications and demonstrated the feasibility of directly converting renewable electricity and CO_2_ into industrial platform chemicals.

#### 5.2.2 Formate

Formate is an important C1 platform molecule that functions as a chemical feedstock, biological hydrogen carrier, and soluble intermediate linking carbon capture with downstream microbial conversion. Because formate can be readily assimilated by several natural and synthetic carbon fixation pathways, it has emerged as an attractive intermediate for integrated C1 biorefineries and hybrid electro-biological production systems (Li et al., 2024).

Tarraran et al. (2023) demonstrated that engineering *Acetobacterium woodii* combined with elevated CO_2_ partial pressure successfully redirected carbon flux toward formate biosynthesis during autotrophic gas fermentation. Besides improving product formation, the study highlighted the importance of reactor pressure as a process variable capable of modulating metabolic fluxes and expanding the range of products obtainable from acetogenic bacteria. Similarly, Oswald et al. (2018) demonstrated that elevated CO_2_ and H_2_ pressures substantially altered the metabolic profile of *Clostridium ljungdahlii*, promoting formate accumulation under autotrophic conditions. Their results provided one of the first demonstrations that high-pressure cultivation can shift acetogenic metabolism toward C1 intermediates, establishing pressure control as an effective strategy for regulating product selectivity during gas fermentation.

#### 5.2.3 Succinate

Succinate is one of the most important C4 platform chemicals produced through microbial CO_2_ assimilation and is widely employed in the manufacture of biodegradable polymers, resins, food additives, pharmaceuticals, and specialty chemicals. Because its biosynthesis incorporates CO_2_ through carboxylation reactions, succinate has long been recognized as a representative example of industrial carbon fixation in microbial biotechnology. Amulya and Mohan (2019) demonstrated that integrating anaerobic fermentation with biological CO_2_ fixation enabled efficient succinate biosynthesis while emphasizing the importance of electron donors and intracellular redox balance in regulating carbon partitioning. Rather than relying solely on microbial strain performance, the study highlighted that optimizing the metabolic environment is equally important for maximizing carbon incorporation into value-added C4 platform chemicals.

#### 5.2.4 Lactate

Lactate is an important C3 platform chemical widely employed in the food, pharmaceutical, cosmetic, and biodegradable polymer industries, particularly as the precursor of polylactic acid (PLA). Despite its broad industrial relevance, lactate is not a native end product of acetogenic metabolism, and its production from CO_2_ remains largely dependent on metabolic engineering strategies (Li et al., 2024).

Metabolic engineering has successfully expanded the metabolic capabilities of *Acetobacterium woodii* by redirecting intracellular carbon flux toward lactate biosynthesis. Deletion of the hydrogenase complex (ΔhydBA/hdcr) altered the intracellular redox balance, promoting efficient redistribution of reducing equivalents from acetate to lactate. Following pathway validation under heterotrophic conditions, the engineered strain demonstrated that C1-derived carbon supplied as methyl-group substrates and CO could be converted into lactate through the Wood–Ljungdahl pathway. These findings establish an important proof-of-concept that acetogenic metabolism can be reprogrammed to produce non-native organic acids and provide a foundation for future development of autotrophic CO_2_-based lactate biomanufacturing through metabolic engineering (Moon et al., 2023).

#### 5.2.5 3 -Hydroxypropionate

3-Hydroxypropionate (3-HP) is a strategic platform chemical widely recognized as a precursor for acrylic acid, acrylonitrile, acrylamide, and biodegradable polymers, making it one of the most attractive non-native products for sustainable carbon biomanufacturing (Liang et al., 2022). Metabolic engineering has successfully expanded the product spectrum of *Cupriavidus necator* beyond its native metabolism by introducing a β-alanine-dependent biosynthetic pathway and eliminating competing metabolic reactions to redirect carbon flux toward 33-HP. Following heterotrophic pathway optimization and identification of key metabolic bottlenecks, the engineered strain was able to produce 3-HP under autotrophic conditions using CO_2_ as the sole carbon source and H_2_ as the electron donor. Although autotrophic production remains at an early stage, this work represents one of the first experimental demonstrations that chemolithoautotrophic *C. necator* can be engineered to synthesize 3-HP directly from CO_2_, establishing an important foundation for future optimization of CO_2_-based production of this industrial platform chemical (Salinas et al., 2022).

#### 5.2.6 Butyrate

*n*-Butyrate is a four-carbon platform chemical widely employed as a precursor for fuels, polymers, specialty chemicals, and microbial chain elongation products. Biological production of butyrate from CO_2_ is particularly attractive because it extends carbon conversion beyond simple C2 intermediates, thereby increasing product value through reverse β-oxidation pathways (Wu et al., 2024). Using continuous microbial electrosynthesis, Jourdin et al. (2019) demonstrated that operational parameters such as CO_2_ loading rate and hydraulic retention time strongly influence product selectivity during bioelectrochemical chain elongation. By steering microbial metabolism beyond acetate, the authors established that mixed microbial biofilms can efficiently upgrade CO_2_ into longer-chain carboxylates, highlighting microbial electrosynthesis as a promising strategy for carbon valorization and sustainable chemical production.

#### 5.2.7 Caproate

*n*-Caproate (hexanoate) is a medium-chain carboxylic acid with growing industrial relevance owing to its applications in biofuels, antimicrobial compounds, specialty chemicals, and feed additives. Compared with short-chain organic acids, caproate possesses a higher market value and greater energy density, making it an attractive target for microbial carbon upgrading. In the same bioelectrochemical platform, Jourdin et al. (2019) demonstrated that microbial chain elongation could be extended from C2 intermediates to medium-chain carboxylates through appropriate reactor operation and biofilm management. Their work established one of the first continuous demonstrations of selective CO_2_ upgrading toward caproate, illustrating the potential of microbial electrosynthesis to diversify the product spectrum beyond conventional acetogenic metabolites.

### 5.3 Lipids and renewable oleochemicals

Lipids constitute an important product class derived from microbial CO_2_ bioconversion owing to their high energy density and broad applications in renewable fuels, oleochemicals, and specialty materials (Gupta et al., 2023). Unlike alcohols and organic acids, lipids can be readily upgraded into biodiesel, sustainable aviation fuel (SAF), lubricants, surfactants, cosmetics, and numerous high-value chemicals. Although fatty acid methyl esters (FAMEs) remain the principal biodiesel products, recent research has increasingly shifted toward advanced oleochemicals and tailored lipid molecules that offer greater economic value and improved commercial prospects. Advances in carbon fixation, metabolic engineering, and synthetic biology have expanded the ability of bacterial platforms to convert inorganic carbon into lipid-based products, reinforcing microbial lipid biosynthesis as a promising route for sustainable carbon utilization (Gupta et al., 2023; Wancura et al., 2025),

#### 5.3.1 Fatty acid methyl esters and lipid-derived biofuels

Fatty acid methyl esters (FAMEs) are the principal constituents of biodiesel and represent one of the earliest lipid-derived products obtained through microbial CO_2_ bioconversion. Beyond conventional biodiesel, microbial lipid metabolism also enables the production of long-chain hydrocarbons and fatty alcohols, considerably expanding the portfolio of renewable fuels and oleochemicals accessible from inorganic carbon.

One of the first demonstrations of chemolithotrophic lipid production from CO_2_ was achieved using *Bacillus cereus* SM1. In addition to high biodiesel yields, CO_2_ fixation supported the simultaneous biosynthesis of C_11_–C_19_ fatty acids and C_11_–C_22_ hydrocarbons, including pentadecane, heptadecane, and nonane. These compounds fall within the carbon-chain range of conventional diesel and aviation fuels, demonstrating that bacterial CO_2_ assimilation can generate multiple classes of fuel molecules rather than biodiesel alone. The study further reported high CO_2_ removal efficiency, highlighting the dual potential of chemolithotrophic bacteria for carbon capture and renewable fuel production (Mishra et al., 2016).

Beyond FAME production, bacterial CO_2_ bioconversion has also been extended to higher-value oleochemicals. Using the halophilic chemolithotroph *Halomonas stevensii* KP163920, thiosulfate-driven CO_2_ fixation enabled the biosynthesis of long-chain fatty alcohols (C_8_–C_27_), with dodecanol and tetradecanol identified as the predominant products. The authors demonstrated efficient CO_2_ utilization together with high biomass productivity under semi-continuous cultivation, highlighting the potential of halophilic bacteria for the sustainable production of fatty alcohols used in detergents, surfactants, lubricant additives, cosmetics, and advanced biofuels (Mishra et al., 2018).

The versatility of bacterial lipid production was further demonstrated through comparative evaluation of different chemolithotrophic platforms. A mixed microbial consortium isolated from activated sludge exhibited superior biodiesel productivity and CO_2_ removal efficiency compared with pure cultures of *Enterobacter cloacae* PP1 and *Pseudomonas putida* SM2. In addition to fatty acid methyl esters, GC–MS analyses identified hydrocarbons ranging from C_9_ to C_32_, including molecules compatible with diesel and kerosene fractions. The superior performance of the mixed consortium was attributed to synergistic metabolic interactions that enhanced inorganic carbon assimilation and broadened the diversity of hydrocarbon products obtained from CO_2_ (Mishra et al., 2019).

Alternative carbon-feeding strategies have also demonstrated considerable potential for microbial lipid biosynthesis. Rather than supplying gaseous CO_2_, *Serratia* sp. ISTD04 utilized dissolved inorganic carbon provided as sodium bicarbonate through a carbon-concentrating mechanism (CCM), enabling efficient synthesis of microbial lipids and C_13_–C_24_ hydrocarbons. Proteomic analyses identified key enzymes involved in carbon fixation and fatty acid biosynthesis, providing molecular evidence that bicarbonate assimilation was effectively coupled to lipid production. Because the resulting hydrocarbons fall within the carbon-chain range of diesel and aviation fuels, this study established bicarbonate as an attractive alternative carbon source for bacterial production of renewable fuel precursors (Kumar et al., 2017).

Building upon this bacterial platform, subsequent work focused on improving biodiesel production through downstream bioprocess engineering. Instead of increasing lipid accumulation, microbial lipids produced by *Serratia* sp. ISTD04 were converted into fatty acid methyl esters using an immobilized lipase from *Pseudomonas* sp. ISTPL3 supported on calcite-impregnated activated biochar. Compared with conventional alkaline transesterification, the enzymatic process substantially increased FAME conversion efficiency while improving catalyst stability and reusability, demonstrating how downstream process optimization can complement microbial CO_2_ bioconversion to enhance the overall sustainability and efficiency of biodiesel production (Kumar and Thakur, 2020).

Collectively, these studies demonstrate that bacterial CO_2_ bioconversion has evolved far beyond the production of conventional biodiesel. Current bacterial platforms can synthesize a diverse portfolio of lipid-derived products, including fatty acids, long-chain hydrocarbons, fatty alcohols, and FAMEs, using both gaseous CO_2_ and dissolved inorganic carbon supplied as bicarbonate. These advances reinforce the potential of bacterial carbon fixation as a sustainable route for producing renewable transportation fuels, sustainable aviation fuel (SAF) precursors, and high-value oleochemicals.

### 5.4 Bioplastics

Bioplastics represent one of the most promising products derived from microbial CO_2_ bioconversion because they combine carbon capture with the production of biodegradable and biocompatible materials. Unlike biofuels, which release carbon following utilization, bioplastics retain fixed carbon in durable products, thereby contributing to long-term carbon storage and supporting circular bioeconomy strategies. Recent advances in autotrophic gas fermentation, metabolic engineering, systems biology, and synthetic biology have substantially improved the ability of hydrogen-oxidizing bacteria to convert CO_2_ into biodegradable polymers, positioning microbial bioplastics as sustainable alternatives to petroleum-derived plastics while expanding opportunities for carbon capture and utilization (Che et al., 2023; Zong et al., 2025).

#### 5.4.1 Polyhydroxyalkanoates (PHAs)

Among microbial bioplastics, polyhydroxyalkanoates (PHAs) have emerged as the most extensively investigated polymer family because of their complete biodegradability, biocompatibility, and thermoplastic properties. Naturally accumulated by numerous bacteria as intracellular carbon and energy storage compounds, PHAs can be synthesized directly from CO_2_ by hydrogen-oxidizing bacteria through autotrophic carbon fixation (Dalton et al., 2022). The family comprises several polymers with distinct physicochemical properties, including poly(3-hydroxybutyrate) (PHB), the most abundant and industrially established homopolymer, and poly(3-hydroxybutyrate-*co*-3-hydroxyvalerate) (PHBV), a copolymer exhibiting lower crystallinity, greater mechanical flexibility, and improved processability for packaging, agriculture, tissue engineering, drug delivery, and other biomedical applications. Recent advances in metabolic engineering, transcriptional regulation, and autotrophic gas fermentation have substantially expanded both the productivity and structural diversity of PHAs obtained through microbial CO_2_ bioconversion (Che et al., 2023; Zong et al., 2025; Li D. et al., 2025).

Kim et al. (2022) demonstrated that enhancing carbon assimilation through the Calvin–Benson–Bassham (CBB) cycle is an effective strategy for improving autotrophic PHB biosynthesis. By co-expressing the global transcriptional regulators CbbR and RegA in *Cupriavidus necator* H16, the authors redirected carbon flux toward polymer accumulation during lithoautotrophic CO_2_/H_2_ cultivation, achieving 1.2 g L^–1^ PHB with 58% of cell dry weight. These findings established transcriptional regulation of carbon fixation as an effective engineering strategy for increasing polymer biosynthesis directly from CO_2_.

Complementary advances were reported by Tang et al. (2022), who shifted the engineering focus from carbon fixation to intracellular polymer accumulation. Comparative transcriptomic analyses identified regulatory genes involved in PHA granule formation and storage, demonstrating that overexpression of the global nitrogen regulator rpoN increased PHB production by 77.5%, whereas overexpression of the granule-associated protein PhaP1 enhanced polymer accumulation by 49.8% under chemoautotrophic CO_2_/H_2_ cultivation. Together, these results demonstrated that engineering intracellular regulatory networks can substantially improve polymer biosynthesis without modifying the central carbon fixation pathway.

More recently, Chang et al. (2025) considerably advanced autotrophic PHA production by integrating process engineering with hydrogen-oxidizing bacterial cultivation. Using *Cupriavidus* sp. CY-1 under optimized autotrophic gas fermentation, the authors achieved 11.87 g L^–1^ PHA, corresponding to 90.6% of cell dry mass, representing one of the highest autotrophic PHA productivities reported to date. The same platform was subsequently adapted for the production of the copolymer PHBV through a two-stage cultivation strategy supplemented with valeric acid, reaching 1.47 g L^–1^ PHBV. These findings demonstrate that recent advances in process engineering have expanded microbial CO_2_ bioconversion beyond conventional PHB production toward a broader portfolio of tailor-made PHAs with improved properties for sustainable packaging, biomedical devices, and other advanced bioplastic applications.

### 5.5 Single-cell protein

The production of proteins and food ingredients through microbial CO_2_ bioconversion represents one of the most promising applications of CCU, as it directly couples carbon sequestration with sustainable food production. Unlike fuels and commodity chemicals, microbial protein remains incorporated into biomass, providing a renewable source of high-quality nutrients while addressing global challenges related to food security, sustainable agriculture, and the environmental impacts of conventional livestock production (Van Peteghem et al., 2022; Alves et al., 2025).

Among the available platforms, single-cell protein (SCP) has attracted particular attention because it enables the direct conversion of inorganic carbon into protein-rich microbial biomass suitable for human food, animal feed, and aquaculture (Rajput et al., 2026). Hydrogen-oxidizing bacteria, particularly *Cupriavidus necator*, have emerged as the leading bacterial chassis owing to their rapid autotrophic growth, balanced amino acid composition, high protein content, and scalability under gas fermentation. Recent advances in cultivation strategies, process engineering, and nutrient optimization have further strengthened the feasibility of producing sustainable protein directly from CO_2_ (Weldon and Euler, 2025; Nath, 2026).

Yang et al. (2021) investigated the influence of nitrogen availability on autotrophic SCP production by *Cupriavidus necator*. Rather than focusing solely on biomass formation, the authors demonstrated that different nitrogen sources substantially affected protein accumulation and cultivation performance while maintaining a stable amino acid profile. The study further highlighted the feasibility of utilizing nitrogen-rich waste streams, such as digestate and urine, as alternative nitrogen sources for sustainable microbial protein production.

Expanding this approach, Ismail et al. (2024) evaluated the influence of environmental cultivation parameters on SCP production by *Cupriavidus necator*. By systematically optimizing temperature and pH, the authors characterized changes in biomass composition, amino acid profile, nucleic acid content, and intracellular carbon storage, providing important insights for improving nutritional quality and process robustness during autotrophic cultivation.

A different strategy was proposed by Li B. et al. (2025), who developed a passive gas-feeding cultivation system for autotrophic SCP production. Instead of conventional continuous gas sparging, the process relied on passive gas transfer to simplify reactor operation, reduce energy requirements, and improve process safety, demonstrating the feasibility of decentralized microbial protein production under resource-limited conditions.

Focusing on industrial implementation, Novak et al. (2025) demonstrated the successful scale-up of autotrophic SCP production using a pilot-scale pressurized gas bioreactor. The study showed that optimizing gas transfer together with chemolithoautotrophic inoculation enabled high-cell-density cultivation while maintaining the nutritional quality of the microbial biomass, representing an important step toward commercial-scale CO_2_-based protein production.

Unlike the previous studies based on direct biomass production, Molitor et al. (2019) proposed a two-stage sequential bioprocess integrating autotrophic acetogenesis with heterotrophic yeast cultivation. In this platform, *Clostridium ljungdahlii* first converted CO_2_ into acetate via the Wood–Ljungdahl pathway, which subsequently served as the carbon source for *Saccharomyces cerevisiae* growth. This integrated approach demonstrated that coupling acetogenic carbon fixation with conventional fermentation can broaden the portfolio of protein ingredients obtainable from microbial CO_2_ bioconversion.

### 5.6 Specialty biomolecules

Specialty biomolecules comprise a diverse group of high-value products derived from microbial CO_2_ bioconversion that extend beyond conventional fuels, commodity chemicals, and structural biopolymers. These compounds include extracellular polysaccharides, biosurfactants, wax esters, and long-chain hydrocarbons with applications in the food, cosmetic, pharmaceutical, lubricant, and specialty chemical industries. Although most of these products remain at an early stage of technological development, recent advances in metabolic engineering, synthetic microbial consortia, gas fermentation, and microbial electrosynthesis have considerably expanded their production from CO_2_, highlighting new opportunities for high-value carbon valorization (Ray et al., 2022; Gupta et al., 2024; Yang M. et al., 2026).

#### 5.6.1 Exopolysaccharides

Exopolysaccharides (EPS) are extracellular biopolymers with broad applications in the food, pharmaceutical, biomedical, and environmental industries. Advances in microbial CO_2_ bioconversion have enabled their sustainable synthesis from inorganic carbon, expanding the portfolio of high-value bio-based materials (Rajapitamahuni et al., 2026). Kumar et al. (2019) demonstrated that *Serratia* sp. ISTD04 produces heteropolysaccharide EPS through bicarbonate-based cultivation. The study combined genomic characterization with process optimization to improve polymer production and identified an EPS predominantly composed of glucose, galactose, mannose, and glucosamine. Functional characterization further demonstrated its excellent bioflocculation performance and dye-removal capacity, highlighting the potential of CO_2_-derived EPS for sustainable environmental and biomedical application.

#### 5.6.2 Wax esters

Wax esters are neutral lipids with high commercial value owing to their excellent lubricating properties, oxidative stability, and biocompatibility. They are widely used in cosmetics, lubricants, pharmaceuticals, and specialty chemicals, making them attractive targets for sustainable CO_2_ biomanufacturing (Pichler et al., 2023). The first two-stage bacterial platform coupling microbial electrosynthesis with aerobic bioconversion to produce wax esters directly from CO_2_ and electricity. In this proof-of-concept system, *Sporomusa ovata* first reduced CO_2_ to acetate through microbial electrosynthesis, which was subsequently converted into long-chain alkyl esters by *Acinetobacter baylyi*. This study demonstrated the feasibility of integrating electrochemical carbon fixation with microbial upgrading, establishing a versatile platform for the production of carbon-neutral oleochemicals, biofuels, and other specialty chemicals (Lehtinen et al., 2017).

#### 5.6.3 Biosurfactants

Biosurfactants are biodegradable amphiphilic biomolecules with broad applications in cosmetics, detergents, pharmaceuticals, food processing, enhanced oil recovery, and environmental remediation owing to their low toxicity and excellent surface-active properties. Among biosurfactant-producing bacteria, *Bacillus* species are considered one of the most important microbial platforms because of their ability to synthesize lipopeptides with high industrial value (Napp et al., 2026). Sundaram and Thakur (2015) demonstrated that the chemolithotrophic bacterium *Bacillus* sp. ISTS2 can simultaneously assimilate CO_2_ and produce lipopeptide biosurfactants under both gaseous CO_2_- and bicarbonate-enriched cultivation. The study confirmed the involvement of a carbon-concentrating mechanism mediated by carbonic anhydrase and RuBisCO and showed that the biosurfactant effectively reduced surface tension and exhibited high emulsifying activity, demonstrating the feasibility of coupling microbial CO_2_ sequestration with the production of high-value surface-active biomolecules

## 6 Current challenges in industrial microbial CO_2_

Despite remarkable advances in microbial CO_2_ bioconversion, its transition from laboratory research to large-scale industrial deployment remains constrained by interconnected biological, engineering, economic, and sustainability challenges. Although diverse microbial platforms have demonstrated the ability to convert CO_2_ into fuels, chemicals, biopolymers, microbial proteins, and other value-added products, process performance is still limited by the physicochemical properties of CO_2_, including its low aqueous solubility and high thermodynamic stability, as well as by constraints associated with microbial physiology, reactor engineering, energy availability, downstream processing, and commercial competitiveness (Kurt et al., 2023; Acuña López et al., 2024).

Overcoming these barriers will require integrated advances in strain engineering, bioprocess intensification, reactor design, renewable energy utilization, digital process control, and sustainability-driven process optimization. Rather than depending on isolated technological breakthroughs, future industrial implementation is expected to emerge from the convergence of biological innovation with engineering, systems integration, and circular carbon management strategies (Sakarika et al., 2024; Jofre et al., 2026). The following sections discuss the principal challenges and emerging strategies shaping the future of microbial CO_2_ biomanufacturing.

### 6.1 CO_2_ mass transfer limitations

Efficient CO_2_ mass transfer remains one of the principal engineering bottlenecks limiting microbial CO_2_ bioconversion. Because CO_2_ exhibits low aqueous solubility and relatively slow diffusion kinetics, substrate availability frequently becomes limiting, particularly in high-cell-density cultures and large-scale bioreactors. Consequently, insufficient gas transfer reduces carbon fixation rates, microbial productivity, and overall process efficiency, making mass transfer one of the key determinants of industrial performance (Li et al., 2024; Carmona-Martínez et al., 2024).

Gas–liquid mass transfer, commonly described by the volumetric mass transfer coefficient (kLa), governs CO_2_ availability during cultivation. Increasing agitation, gas flow rate, operating pressure, or gas recirculation generally improves CO_2_ transfer but simultaneously increases energy demand, shear stress, and operating costs. Therefore, reactor optimization requires balancing gas-transfer efficiency with microbial physiology, process economics, and operational stability rather than maximizing a single engineering parameter (Hajinajaf et al., 2024; Lindeque and Woodley, 2026).

Recent advances in reactor engineering have focused on overcoming these limitations through process intensification strategies, including membrane-based gas delivery, hollow-fiber bioreactors, microbubble generation, pressurized cultivation, and computational fluid dynamics (CFD)-assisted reactor optimization. More recently, concepts associated with Bioprocessing 4.0, such as digital process monitoring, predictive control, advanced sensors, and data-driven reactor operation, have emerged as promising tools for dynamically optimizing gas transfer and process performance under industrial conditions (Isoko et al., 2024). Future improvements are therefore expected to rely not only on enhanced reactor hydrodynamics but also on the integrated co-design of microbial metabolism and bioreactor architecture, enabling higher carbon utilization efficiency while minimizing energy consumption and operating costs (Wu et al., 2024; Sadvakasova et al., 2026).

### 6.2 Biological constraints and comparison with non-biological CO_2_ conversion

Biological limitations remain a major obstacle to efficient microbial CO_2_ bioconversion. Although microorganisms possess diverse carbon fixation pathways, these metabolic networks evolved to maximize cellular fitness under natural environments rather than carbon conversion efficiency under industrial conditions. Consequently, microbial performance is constrained not only by relatively low carbon assimilation rates but also by limited metabolic robustness, physiological adaptability, and product tolerance, restricting the volumetric productivities required for the large-scale production of fuels, chemicals, polymers, and microbial proteins (Bae et al., 2024; Li et al., 2026).

Carbon assimilation is governed by multiple interconnected physiological processes, including enzyme kinetics, ATP availability, reducing-equivalent generation, redox homeostasis, cofactor balance, oxygen sensitivity, and intracellular carbon partitioning. Fixed carbon must simultaneously support biomass formation, cellular maintenance, energy conservation, stress responses, and product synthesis, generating inherent trade-offs between microbial growth and metabolite production. These physiological constraints become increasingly pronounced under industrial conditions, where microorganisms are exposed to high gas partial pressures, continuous cultivation, substrate fluctuations, and product inhibition (Zhao et al., 2021; Xu P. et al., 2024; Chen et al., 2026).

Future engineering strategies increasingly combine systems metabolic engineering with genome-scale engineering, CRISPR-based genome editing, adaptive laboratory evolution (ALE), and synthetic metabolism to overcome these biological constraints. Rather than optimizing individual genes or enzymes, next-generation engineering strategies aim to redesign cellular metabolism through dynamic carbon flux regulation, cofactor balancing, elimination of competing pathways, and the development of robust programmable microbial chassis capable of maintaining stable productivity under industrial operating conditions (Yilmaz et al., 2022; Wenk et al., 2022; Kim et al., 2026).

The relationship between microbial growth rate and biomanufacturing performance further illustrates the biological constraints associated with engineered production hosts. *Vibrio natriegens*, a fast-growing bacterial chassis that has recently attracted considerable interest for biomanufacturing, combines rapid growth with broad substrate utilization and an expanding synthetic-biology toolbox. Its high growth rate can translate into substantial productivity gains; for example, engineered *V. natriegens* reached a 2,3-butanediol productivity of 3.44 g L^–1^ h^–1^, compared with 1.19 g L^–1^ h^–1^ reported for *Escherichia coli*. However, this productivity advantage does not necessarily translate into higher titers or yields, which frequently remain lower than those achieved with established production hosts. Rapid biomass formation may also intensify oxygen- and mass-transfer limitations at high cell densities, potentially increasing overflow metabolism and carbon diversion. Thus, *V. natriegens* exemplifies both the potential and the remaining bottlenecks of fast-growing engineered chassis: high specific growth and productivity must ultimately be balanced with carbon yield, product titer, metabolic stability, and reactor-scale mass-transfer capacity (Lima et al., 2024).

The integration of computational biology, systems-level omics, and automated Design–Build–Test–Learn (DBTL) workflows is further accelerating strain development by enabling predictive identification of metabolic bottlenecks and engineering targets. Combined with adaptive laboratory evolution and biofoundry platforms, these approaches are expected to substantially improve microbial robustness, carbon utilization efficiency, and process reliability, facilitating the transition from laboratory-scale proof-of-concept studies to industrial microbial CO_2_ biomanufacturing (Heffernan et al., 2024; Xu P. et al., 2024; Ramos et al., 2026).

These biological constraints become particularly relevant when microbial CO_2_ bioconversion is benchmarked against non-biological conversion technologies. Thermochemical routes generally provide high reaction rates and are compatible with large-scale continuous processing, although they may require elevated temperatures, pressures, and substantial energy inputs. Electrochemical CO_2_ conversion can achieve substantially higher reaction rates than microbial electrosynthesis and can be directly coupled to renewable electricity, but its overall performance remains influenced by cell voltage, energy efficiency, catalyst stability, carbon losses, and product-separation requirements. Photochemical approaches offer the possibility of directly harvesting light energy, yet their broader implementation remains constrained by comparatively low energy-conversion efficiencies and scale-up requirements (Grim et al., 2026; Khiarak et al., 2026; Meshkani et al., 2027).

Microbial platforms present a different set of trade-offs. Their comparatively mild operating conditions and enzyme-mediated reactions enable high product selectivity and direct synthesis of structurally complex multicarbon compounds, potentially reducing the number of conversion and upgrading steps. However, these advantages are counterbalanced by lower reaction rates and volumetric productivities, biological maintenance requirements, gas-liquid mass-transfer limitations, electron or H_2_ delivery, product inhibition, and carbon partitioning between biomass, target products, and metabolic by-products. Therefore, biological routes should not be considered inherently more sustainable or technologically advantageous than non-biological alternatives. Rather, technology selection requires a system-level comparison of productivity, energy demand, carbon efficiency, process complexity, scalability, product selectivity, and downstream requirements (Grim et al., 2026; Meshkani et al., 2027).

### 6.3 Scale-up, process integration and industrial deployment

The translation of microbial CO_2_ bioconversion from laboratory research to commercial deployment remains one of the greatest challenges facing the field. Although laboratory-scale systems often achieve high carbon conversion efficiencies under tightly controlled conditions, industrial implementation requires maintaining stable productivity under heterogeneous operating environments characterized by gas concentration gradients, imperfect mixing, fluctuating feed composition, contamination risks, and increasing operational complexity. Consequently, successful scale-up depends on the simultaneous optimization of reactor hydrodynamics, gas–liquid mass transfer, microbial robustness, and process control rather than on improvements in microbial performance alone (Malusare et al., 2023; Li et al., 2024).

Beyond reactor scale-up, industrial implementation requires efficient integration of upstream cultivation, gas conditioning, nutrient supply, continuous operation, and downstream processing (Xu H. et al., 2024). Maintaining long-term process stability also depends on robust microbial chassis capable of tolerating fluctuating industrial conditions while sustaining high productivity. Recent developments in Bioprocessing 4.0, including digital twins, advanced sensors, real-time monitoring, and predictive process control, are creating new opportunities to improve operational robustness, facilitate continuous cultivation, and optimize large-scale bioprocesses (Roy et al., 2022; Isoko et al., 2024; Xu P. et al., 2024).

Future industrial deployment is expected to rely increasingly on integrated carbon utilization systems, where microbial CO_2_ bioconversion is coupled with carbon capture, renewable electricity, green hydrogen production, and complementary CCUS technologies. Rather than functioning as isolated conversion processes, microbial biorefineries are likely to operate within integrated carbon hubs that promote industrial symbiosis, resource efficiency, and circular carbon utilization. The integration of Power-to-X technologies, direct air capture (DAC), and renewable energy infrastructure may further improve operational flexibility while enabling carbon valorization according to regional energy availability and market demands (Li et al., 2024; Wesche and Skjølsvold, 2025; Huerta-Rosas et al., 2026).

Despite rapid technological progress, technology readiness levels (TRLs) still differ substantially among microbial CO_2_ conversion platforms. While gas fermentation has already demonstrated commercial feasibility in selected applications, microbial electrosynthesis, hybrid electro-biological systems, and several emerging carbon utilization technologies still require advances in reactor engineering, long-term operational stability, process integration, and industrial validation before large-scale commercialization becomes feasible. Accelerating this transition will require closer integration between biotechnology, chemical engineering, digital manufacturing, renewable energy systems, and industrial carbon management (Jofre et al., 2026; Bose et al., 2026; Mohamadi, 2026).

### 6.4 Integrated techno-economic and environmental assessment

Techno-economic assessment (TEA) and life cycle assessment (LCA) are essential for evaluating the commercial and environmental feasibility of microbial CO_2_ bioconversion. TEA integrates biological performance with capital and operating expenditures, energy demand, downstream processing, and product value, whereas LCA evaluates greenhouse gas emissions, energy demand, and resource consumption across the production chain. Their combined application during early process development can identify major technological bottlenecks and prioritize engineering strategies with greater potential for industrial implementation (Müller et al., 2020; Zimmermann et al., 2022; Vickram et al., 2025).

Process economics are particularly sensitive to gas-transfer efficiency, electricity and H_2_ requirements, CO_2_ sourcing and capture, downstream processing, and product value. Renewable electricity and green H_2_ can represent major operating costs in hydrogenotrophic and electro-biotechnological platforms, while CO_2_ capture costs depend strongly on source and concentration (Enzmann et al., 2021; Almomani et al., 2023). Downstream recovery can further dominate costs for dilute fermentation products or intracellular compounds. Consequently, commercial viability depends on the combined optimization of titer, productivity, selectivity, recovery efficiency, and energy demand rather than carbon conversion alone. Higher-value products such as PHAs, microbial proteins, biosurfactants, specialty chemicals, and medium-chain carboxylates may therefore provide more favorable near-term opportunities than commodity products requiring very high volumetric productivity and low production costs (Vlaeminck et al., 2023; Jean and Brown, 2024; Li et al., 2026).

Environmental performance likewise cannot be inferred from the use of captured CO_2_ alone. Claims of carbon-neutral or net carbon-negative production require a system-level carbon balance accounting for CO_2_ origin and capture, electricity carbon intensity, H_2_ or reducing-equivalent production, process energy demand, downstream separation, and the fate and lifetime of carbon in the final product. Coupling microbial conversion with low-carbon electricity, low-carbon H_2_, and appropriately sourced CO_2_ can improve greenhouse gas mitigation potential, but the magnitude of this benefit remains system-dependent (Zimmermann et al., 2022; Wang Y. et al., 2025; Wang Z. et al., 2025). Similar constraints apply to alternative CO_2_-conversion technologies. For example, photocatalytic CO_2_ reduction using metal-organic frameworks can generate CO, CH_4_, CH_3_OH, and HCOOH but remains constrained by charge recombination, quantum efficiency, product selectivity, catalyst stability, recyclability, and scale-up (Goyal et al., 2026). Thus, biological and non-biological CO_2_-conversion technologies should be assessed using consistent system boundaries and life-cycle criteria rather than assuming environmental superiority from CO_2_ utilization itself (Wesche and Skjølsvold, 2025).

Future commercialization will therefore require integrated TEA-LCA frameworks that consider carbon intensity, fossil-carbon displacement, product lifetime, life-cycle greenhouse gas emissions, and economic performance alongside conventional bioprocess metrics. Regulatory frameworks, carbon pricing, renewable-energy availability, and regional infrastructure will further influence technology selection and deployment. Embedding these assessments throughout strain and process development should facilitate the identification of platform–product combinations capable of progressing from laboratory-scale demonstration toward economically viable and environmentally sustainable CO_2_ biomanufacturing (Rodin et al., 2022; Sakarika et al., 2024; Chlela and Selosse, 2025; Jofre et al., 2026; Gonçalves et al., 2026).

### 6.5 Critical perspective

Despite remarkable advances in microbial CO_2_ bioconversion, a substantial gap remains between laboratory innovation and industrial implementation. Much of the current research continues to prioritize improvements in individual biological components, including enzymes, metabolic pathways, and microbial chassis, while comparatively less attention has been devoted to the integration of microbial physiology with reactor engineering, process intensification, downstream processing, and industrial system design. Consequently, further gains in carbon fixation efficiency alone are unlikely to overcome the technological and economic barriers that currently limit large-scale commercialization.

From an industrial perspective, the future of microbial CO_2_ bioconversion should not be driven by the search for a universal microbial platform or a single optimal carbon fixation pathway. Instead, industrial success will likely depend on the development of flexible microbial manufacturing platforms in which microbial hosts, carbon fixation pathways, reactor configurations, and downstream processing strategies are selected according to the desired product portfolio, regional carbon sources, renewable energy availability, and market demand. This platform-oriented approach recognizes that no single biological system is expected to maximize productivity, carbon efficiency, product diversity, and process economics simultaneously across all applications.

Another important shift concerns how emerging technologies are evaluated. Academic research has traditionally emphasized carbon fixation efficiency and theoretical metabolic performance as the principal indicators of technological progress. However, successful industrial deployment will increasingly depend on broader performance metrics, including volumetric productivity, process robustness, operational flexibility, scalability, downstream processing efficiency, techno-economic competitiveness, and environmental sustainability. Integrating these criteria from the earliest stages of strain and process development will be essential for accelerating technology maturation and reducing the gap between proof-of-concept studies and commercial implementation.

Ultimately, microbial CO_2_ bioconversion should be regarded not as a standalone carbon utilization technology, but as one component of an integrated carbon management strategy. In our view, its greatest industrial potential lies in the development of modular biomanufacturing platforms capable of converting captured carbon into diversified product portfolios according to market demand, regional infrastructure, and renewable energy availability. Such an approach shifts the focus from maximizing carbon fixation alone to designing flexible, economically viable, and environmentally sustainable production systems. Rather than competing directly with petrochemical manufacturing, microbial CO_2_ bioconversion is more likely to complement existing industrial processes by supplying selected fuels, chemicals, materials, and proteins where biological conversion provides clear technical or environmental advantages.

## 7 Concluding remarks

The accelerating climate crisis and the transition toward carbon-neutral manufacturing have strengthened the relevance of microbial CO_2_ bioconversion as a strategy for sustainable carbon utilization. Beyond conventional approaches centered on emission mitigation or carbon storage, microbial systems offer the possibility of coupling carbon fixation with biological manufacturing, enabling CO_2_ to serve as a renewable carbon feedstock to produce fuels, chemicals, biomaterials, proteins, and other value-added products. The evidence synthesized in this review indicates that this concept is progressively evolving from a predominantly fundamental research topic toward a broader technological framework connecting microbiology, biotechnology, environmental science, engineering, and sustainable manufacturing.

This transition is also reflected in the bibliometric landscape. The sustained expansion of scientific output, diversification of disciplinary contributions, development of international collaboration networks, and strong interconnection among the major thematic clusters demonstrate that microbial CO_2_ bioconversion is no longer organized around carbon fixation alone. Instead, contemporary research increasingly integrates microbial diversity, carbon assimilation, metabolic pathways, molecular engineering, energy conservation, and product formation. This convergence is particularly important because improving biological carbon fixation in isolation is unlikely to be sufficient for industrial implementation; microbial performance must ultimately be integrated with efficient energy supply, gas–liquid mass transfer, reactor operation, product recovery, and process economics.

At the biological level, advances in natural and synthetic carbon fixation pathways, metabolic engineering, synthetic biology, systems biology, and programmable microbial chassis are progressively expanding the range and efficiency of CO_2_-derived products. At the process level, gas fermentation, microbial electrosynthesis, bioelectrochemical systems, synthetic microbial consortia, renewable hydrogen integration, and data-driven bioprocess optimization provide complementary routes for translating these biological capabilities into scalable production platforms. Artificial intelligence and genome-scale modeling may further accelerate this transition by enabling more rational strain design, pathway optimization, and integration of biological and process-level variables.

Nevertheless, substantial barriers still separate laboratory-scale performance from economically competitive industrial deployment. Low CO_2_ solubility and mass-transfer rates, energetic requirements for carbon fixation, limitations in carbon conversion efficiency and product selectivity, reactor scalability, downstream processing, and dependence on low-carbon energy remain major constraints. Consequently, future development should move beyond maximizing isolated biological metrics and adopt an integrated strain–pathway–reactor–process perspective. Techno-economic assessment and life cycle assessment should therefore accompany technology development from early stages, allowing biological improvements to be evaluated in terms of their actual contribution to productivity, carbon efficiency, energy demand, environmental performance, and economic feasibility.

The next stage of the field will likely depend less on a single breakthrough than on the strategic convergence of complementary technologies. Programmable microbial chassis, next-generation carbon fixation pathways, genome-scale engineering, artificial intelligence, renewable electricity, green hydrogen, intensified bioreactors, and integrated carbon biorefineries could collectively enable more efficient and flexible CO_2_ conversion platforms. Importantly, future progress should prioritize not only higher fixation rates, but also carbon efficiency, product specificity, process robustness, scalability, and demonstrable environmental benefit, thereby narrowing the persistent gap between proof-of-concept studies and industrial implementation.

Ultimately, microbial CO_2_ bioconversion should be viewed not simply as an alternative route for carbon conversion, but as an emerging platform for carbon circularity and sustainable biomanufacturing. Its long-term impact will depend on the ability to connect fundamental microbial carbon metabolism with engineering, renewable energy, digital technologies, and industrial process design. If these dimensions can be successfully integrated, microbial systems may contribute to transforming CO_2_ from an unavoidable waste stream into a renewable feedstock for producing chemicals, materials, fuels, and other bioproducts, while supporting broader strategies for industrial decarbonization and the circular bioeconomy.

## Data Availability

The original contributions presented in the study are included in the article/Supplementary material, further inquiries can be directed to the corresponding authors.
